# Cytokinin Metabolism of Pathogenic Fungus *Leptosphaeria maculans* Involves Isopentenyltransferase, Adenosine Kinase and Cytokinin Oxidase/Dehydrogenase

**DOI:** 10.3389/fmicb.2017.01374

**Published:** 2017-07-21

**Authors:** Lucie Trdá, Monika Barešová, Vladimír Šašek, Miroslava Nováková, Lenka Zahajská, Petre I. Dobrev, Václav Motyka, Lenka Burketová

**Affiliations:** ^1^Institute of Experimental Botany, The Czech Academy of Sciences Prague, Czechia; ^2^Department of Biochemistry and Microbiology, Institute of Chemical Technology Prague, Czechia

**Keywords:** cytokinin, *Leptosphaeria maculans*, isopentenyltransferase, adenosine kinase, cytokinin oxidase/dehydrogenase, zeatin *cis*/*trans* isomerase

## Abstract

Among phytohormones, cytokinins (CKs) play an important role in controlling crucial aspects of plant development. Not only plants but also diverse microorganisms are able to produce phytohormones, including CKs, though knowledge concerning their biosynthesis and metabolism is still limited. In this work we demonstrate that the fungus *Leptosphaeria maculans*, a hemi-biotrophic pathogen of oilseed rape (*Brassica napus*), causing one of the most damaging diseases of this crop, is able to modify the CK profile in infected *B. napus* tissues, as well as produce a wide range of CKs *in vitro*, with the *cis*-zeatin derivatives predominating. The endogenous CK spectrum of *L. maculans in vitro* consists mainly of free CK bases, as opposed to plants, where other CK forms are mostly more abundant. Using functional genomics, enzymatic and feeding assays with CK bases supplied to culture media, we show that *L. maculans* contains a functional: (i) isopentenyltransferase (IPT) involved in *c*Z production; (ii) adenosine kinase (AK) involved in phosphorylation of CK ribosides to nucleotides; and (iii) CK-degradation enzyme cytokinin oxidase/dehydrogenase (CKX). Our data further indicate the presence of *cis*–*trans* isomerase, zeatin *O*-glucosyltransferase(s) and *N*^6^-(Δ^2^-isopentenyl)adenine hydroxylating enzyme. Besides, we report on a crucial role of LmAK for *L. maculans* fitness and virulence. Altogether, in this study we characterize in detail the CK metabolism of the filamentous fungi *L. maculans* and report its two novel components, the CKX and CK-related AK activities, according to our knowledge for the first time in the fungal kingdom. Based on these findings, we propose a model illustrating CK metabolism pathways in *L. maculans*.

## Introduction

Phytohormones play essential roles in coordination of plant growth, development and stress responses. Different plant-associated microorganisms aim to deregulate phytohormone levels in host plants, either by direct *de novo* hormone production or by affecting their biosynthesis or metabolism in the host (Jameson, 2000; Tsavkelova et al., 2006; Kazan and Lyons, 2014; Chanclud and Morel, 2016). Among the phytohormones, CKs are key regulators of plant development by promoting cell division, or cytokinesis, mainly in plant roots and shoots. CKs primarily control cell growth and differentiation; regulate apical dominance, axillary bud growth, leaf senescence, seed dormancy or nutritional signaling. They are also involved in responses to different environmental stimuli, such as biotic or abiotic stresses (Sakakibara, 2006).

Cytokinins occurring in plants are adenine derivatives with an isoprenoid side chain coupled to the *N*^6^-terminus. The isoprenoid CKs occur as four types: *N*^6^-(Δ^2^-isopentenyl)adenine (iP); and zeatin-type CKs with a hydroxylated isoprenoid side chain, which include *cis*-zeatin (*c*Z), *trans*-zeatin (*t*Z) and dihydrozeatin (DHZ). In plants, *t*Z and iP are the main biologically active CKs, while *c*Z is less active and related to senescence and/or to growth limitations under adverse conditions (Gajdošová et al., 2011; Schäfer et al., 2015). The first and rate-limiting step in biosynthesis of isoprenoid CKs consists of the attachment of an isoprenoid side chain to the adenine nucleotide catalyzed by isopentenyltransferases (IPTs). Plants use two different types of IPTs, depending on the adenine-containing substrate. The first involves adenylate IPTs (IPT; EC 2.5.1.112) that use either ATP, ADP or AMP as a substrate to form iP nucleotides (Kakimoto, 2001; Takei et al., 2001). IPTs of the second type involve tRNA-IPTs (EC 2.5.1.75) that isoprenylate adenosine moieties (A_37_), 3′adjacent to the anticodon of certain tRNAs (Miyawaki et al., 2006). Isoprenylation of tRNAs increases the fidelity of protein biosynthesis stabilizing codon-anticodon binding (Konevega et al., 2006). Except in Archaea, tRNA-IPTs and modified tRNAs are present in all organisms (Persson et al., 1994; Frébort et al., 2011). It is believed that tRNA breakdown partially contributes to the CK pool in plants. Adenylate IPTs exist in multiple copies in all flowering plants and are mainly responsible for the synthesis of iP- and *t*Z-type CKs (Miyawaki et al., 2006). In Arabidopsis, tRNA-IPTs exclusively synthesize *c*Z-type CKs (Miyawaki et al., 2006). Additionally, it was also proposed that *t*Z could be formed from *c*Z via *cis*–*trans* isomerase as described on the partially-isolated enzyme from seeds of *Phaseolus vulgaris* (Bassil et al., 1993), though its activity *in vivo* was not proved. The iP-type nucleotides are further hydroxylated by CK-specific cytochrome P450 monooxygenases leading to *t*Z riboside di- or triphosphates (Takei et al., 2004). The activation of nucleotides to free CK bases is achieved by a one-step reaction catalyzed by LONELY GUY (LOG) (Kurakawa et al., 2007), or by a two-step reaction catalyzed by 5′-ribonucleotide phosphohydrolase and adenosine nucleosidase (Sakakibara, 2006).

CKs can be further modified at the purine ring, or at the side-chain hydroxyl group, undergoing mainly glycosylation, aminoacylation or phosphorylation. The CK free bases are considered as biologically-active forms in plants, while CK nucleosides, nucleotides or glucosides, are mostly less active or inactive (Mok and Mok, 2001). CKs can be glucosylated, most frequently at the *N*7 and *N*9 positions, although *N*3 glucosides have also been detected in several plants (Letham et al., 1975). Zeatin and DHZ-type CKs can also undergo *O*-glucosylations catalyzed by *O*-glucosyltransferases (Frébort et al., 2011). Whereas *N*-glucosides are mostly considered as stable CK deactivation products, *O*-glucosides serve as storage forms in plants (Frébort et al., 2011). CKs can be irreversibly degraded by CK oxidase/dehydrogenases (CKXs) that cleave the *N*^6^ side chain. Plants possess distinct CKX isoforms, differing in their cellular localization and substrate specificity (Frébort et al., 2011). In general, the substrates for CKX are not only CK free bases, but also ribosides, *N*-glucosides and nucleotides (Galuszka et al., 2007). CK nucleotides are either synthesized *de novo* or recycled from their corresponding nucleosides or bases. In plants, AK (EC 2.7.1.20) recycles CK ribosides to riboside 5′-monophosphates (Kwade et al., 2005; Schoor et al., 2011). In addition, CK free bases can be directly recycled to nucleotides by the activity of adenine phosphoribosyltransferases (APT), as for AtAPT1 of Arabidopsis (Zhang et al., 2013).

Manipulation of the CK levels of a host plant upon infection has been reported in different pathosystems in different ways (Kazan and Lyons, 2014). Some invaders affect the CK biosynthesis/metabolism/signaling of the host, such as *Pseudomonas syringae* effector HopQ1 inducing host CK signaling to suppress host defenses (Hann et al., 2014), while others can directly synthesize CKs *de novo*. Infections with gall-forming bacteria are associated with increased CK levels in the infection site (Jameson, 2000). For instance, *Agrobacterium tumefaciens* bacteria deliver *ipt* gene (*tmr*) into the host genome, leading to a massive production of *t*Z (Akiyoshi et al., 1984), while *Rhodococcus fascians* produces a mixture of CKs (Pertry et al., 2009). In addition, CK-producing fungi have been reported, for instance: *Magnaporthe oryzae* (Jiang et al., 2013; Chanclud et al., 2016), *Pyrenopeziza brassicae* (Murphy et al., 1997), *Cladosporium fulvum* (Murphy et al., 1997), *Ustilago maydis* (Bruce et al., 2011) or *Claviceps purpurea* (Hinsch et al., 2015). On the other hand, in *Colletotrichum graminicola*-infected maize leaves, green islands rich in CKs are formed at infection sites (Behr et al., 2012), although this fungus cannot synthesize CKs *de novo*. The importance of CKs for pathogenic virulence has recently been reported for some fungi (Chanclud et al., 2016; Hinsch et al., 2016). For instance, the CKs of *M. oryzae* act as effectors dampening the host’s defenses (Chanclud et al., 2016). CKs are also involved in interactions with beneficial microorganisms. The mycorrhizal fungi *Rhizopogon ochraceorubens* or *R. roseolus* produce CKs (Miller, 1967; Crafts and Miller, 1974). CKs can also act as signals to activate plant responses, as in mycorrhiza (Miransari et al., 2014).

Pathways for the biosynthesis and metabolism of CKs were extensively studied in plants and plant-associated bacteria (Frébort et al., 2011). The knowledge for the fungal kingdom was missing until very recently, when genes involved in CK biosynthesis were reported in the pathogenic fungi *C. purpurea* (Hinsch et al., 2015, 2016) and *M. oryzae* (Chanclud et al., 2016). CK production in *C. purpurea* involves IPTs, cytochrome P450 and LOG activities (Hinsch et al., 2015, 2016), similarly to plants or bacterial genes (Frébort et al., 2011), and a unique bi-functional enzyme CpIPT-LOG containing both IPT and LOG domains (Hinsch et al., 2015). However, the knowledge on further components of fungal CK metabolism involving interconversions and de/inactivation steps is still missing, which motivated this study.

*Leptosphaeria maculans* is a fungal plant extracellular pathogen belonging to the *Dothideomycetes*. This fungus mainly infects *Brassica* crops. In oilseed rape (*Brassica napus*), *L. maculans* causes phoma stem canker (so-called blackleg), the most damaging disease of this crop in Australia, Canada, and Europe (West et al., 2001; Howlett, 2004). The infection cycle begins with ascospores germinating on the leaf surface, invading the cotyledons and younger leaves via stomata or wounds. The fungus grows in the extracellular space and initially colonizes the tissue as a biotroph. Behind the hyphal front the fungus becomes necrotrophic, invading and killing plant cells, and forming asexual fruiting bodies (pycnidia) in the dead tissue. This stage could be described as the primary leaf infection: forming grayish necroses. The hyphal front spreads down the petiole in an endophytic and asymptomatic manner, finally reaching the stem cortex and causing black/brown blackleg necrotic lesions (Hammond and Lewis, 1987; West et al., 2001). Due to the switch in its lifestyle, the fungus is considered as a hemibiotroph.

In this study, the CK metabolism of oilseed rape and its modifications upon infection with *L. maculans* were investigated. We report here that the CK profile is altered in infected plant tissue and that *L. maculans* may contribute to a change of the CK pool. Furthermore, CK production, biosynthetic and transformation routes were studied in the *L. maculans* mycelia grown *in vitro*. Using functional genomics and feeding assays, we show that *L. maculans* contains functional *IPT* and *AK* genes, encoding enzymes that catalyze the formation of *c*Z and the phosphorylation of CK ribosides into nucleotides, respectively. Furthermore, using enzymatic assays, we demonstrate the activity of the CK degradation enzyme CKX, according to our knowledge for the first time in the fungal kingdom. We also suggest here the presence of *cis*–*trans* isomerase, and zeatin *O*-glucosyltransferase(s) activities, as well as the distinct metabolic fate of iP and both zeatin isomers in *L. maculans*. Based on these findings, a model of CK metabolism pathways in *L. maculans* is proposed.

## Materials and Methods

### Fungal and Plant Cultivation

The *L. maculans* isolate JN3, also referred to as v23.1.3 (Balesdent et al., 2001), and prepared JN3-derived transformants were used. The *Lm* isolate JN3 was used given its sequenced and annotated genome (Rouxel et al., 2011) and its use in multiple previous studies on *L. maculans* (Fudal et al., 2007; Šašek et al., 2012b). For gene expression and hormone production, *L. maculans* was cultivated in a liquid Gamborg B5 medium (Duchefa, G0210, Haarlem, The Netherlands), supplemented with 3% (w/v) sucrose and buffered with 10 mM MES (pH 6.8) at a concentration of 10^5^ conidia/ml in 100 ml cultures in Erlenmeyer flasks. Cultures were kept at 26°C, in the dark and at constant shaking of 130 rpm in an orbital shaker (JeioTech, Seoul, Korea). Seven day old mycelium was used for feeding tests. Sporulation was done according to (Ansan-Melayah et al., 1995). The 7-day-old mycelium, grown on V8 juice agar plates, was mashed-up and distributed on new V8 juice agar plates, sealed with Micropore^TM^ (3M) tape, and kept under light in a cycle of 14 h of day (150 μE m^-2^ s^-1^, 22°C) and 10 h of night (19°C) at 70% relative humidity in a climatic chamber. Conidia were washed once with distilled water after harvesting, diluted to 10^8^ conidia/ml, and stored at -20°C for a maximum period of 12 months. For growth tests, mycelium in a liquid culture was collected and weighed at 10 days. For growth tests on solid media, mycelial disks of 7-day-old *L. maculans* were transferred onto V8 juice agar plates covered by cellophane. Growth rate of the fungus was assessed by measuring the diameter of the radially grown mycelium. Otherwise, the mycelium was scraped off and weighed. *B. napus* plants of cultivar Eurol were grown in perlite nourished with Steiner’s nutrient solution (Steiner, 1984) in a cycle of 14 h of day (150 μE m^-2^ s^-1^, 22°C) and 10 h of night (19°C) at 50% relative humidity in a cultivation room.

### Antifungal Assay

The *L. maculans* isolate JN3 (Balesdent et al., 2001) was transformed with a pCAMBgfp construct (Sesma and Osbourn, 2004) carrying the *sGFP* gene, following the protocol according to (Šašek et al., 2012a). Conidia of JN3-GFP were grown sterile in Gamborg B5 medium (Duchefa) supplemented with 0.3% (w/v) sucrose and 10 mM MES (pH 6.8) at the final concentration of 2500 conidia per well of black 96-well plate (Nunc^®^). Plates covered with lids and sealed with Parafilm were incubated in darkness at 26°C. Fluorescence was measured using a Tecan F200 fluorescence reader (Tecan, Männedorf, Switzerland) equipped with a 485/20 nm excitation filter and 535/25 nm emission filter. Eight wells for each treatment were measured.

### Fungal Treatment

Cytokinins *N*^6^-(Δ^2^-isopentenyl)adenine [iP; 6-(γ,γ-Dimethyl allylamino)purine, Sigma, St. Louis, MO, United States), *trans*-zeatin [*t*Z; 6-((E)-4-hydroxy-3-methylbut-2-enylamino)purine, Apex Organics, Leicester, United Kingdom] and *cis*-zeatin [*c*Z; 6-((Z)-4-hydroxy-3-methylbut-2-enylamino)purine, Olchemin, Olomouc, Czechia] were dissolved in 66% (v/v) ethanol (EtOH) as a 1 mM stock solution and stored at -20°C. Stocks prepared for antifungal assays were 100 mM and included the aforementioned CK, *N*^6^-benzyladenine (BA, Sigma, St. Louis, MO, United States) and kinetin (Duchefa, Haarlem, The Netherlands). For CK conversion assays, 7-day-old liquid culture of *L. maculans* was treated with 1 μM CK or mock (66% EtOH, v/v) treatment. The final concentration of ethanol was 0.06% (v/v). For the boiling treatment, an Erlenmeyer flask with sterile *L. maculans* culture was heated to 100°C and kept boiling for 6 min, 1 h prior to the experiment to enable cooling down before treatment with CK. Control media without the *L. maculans* were kept 7–8 days in the assay conditions until sampling. Mycelium and medium from 10-ml aliquots sampled in a time course was harvested by vacuum filtration over a paper filter.

### Pathogen Infection

Infection with *L. maculans* was carried out on the cotyledons of 14-day old plants. For hormone analysis upon infection, each half of a cotyledon was first punctured by a sterile needle, then covered by 10 μl of conidial aqueous suspension (10^6^ conidia/ml) or distilled water (mock). At 7 and 9 days after inoculation, a 4 mm circle of tissue around the puncture, and a 1 mm thick band neighboring, but outside, the lesion borders was sampled, respectively. For evaluation of the virulence of transformants, plants were inoculated by infiltration of a conidial suspension (10^5^ conidia/ml) or distilled water (mock) into the cotyledons using a 1 ml plastic syringe. True leaves were removed from 14 day- to 21 day-old plants to avoid cotyledon senescence. At least 12 different plants were used per condition. Leaves were assessed for lesions 10 days after inoculation. The relative lesion area was calculated based on lesion area and leaf area measured by image analysis using APS Assess 2.0 software (American Phytopathological Society, St. Paul, MN, United States). The average relative lesion area for control plants was set to 100%.

### Preparation and Characterization of Silenced *L. maculans* Lines

A 500-pb region of the target gene Lm*IPT* or Lm*AK* was amplified from the cDNA using a proof-reading Pfu polymerase (Thermo Fisher Scientific, Waltham, MA, United States) and attB1- and attB2-tailed primers. The primers for *LmIPT* were as follows: F: GGGGACAAGTTTGTACAAAAAAGCAGGCTAGTCAAGCCTCTAATCACCA, R: GGGGACCACTTTGTACAAGAAAGCTGGGT-GTTGGATCTTTCGTCGCT, and for *LmAK*: F: GGGGACAAGTTTGTACAAAAAAGCAGGCTCAGGGTGTTGGTGATGAG, R: GGGGACCACTTTGTACAAGAAAGCTGGGT-GAGAGGTTGAGGATGAATGG. The fragment was cloned into a pDONR-Zeo vector (Invitrogen, Carlsbad, CA, United States) using BP clonase (Invitrogen), then recombined by LR clonase (Invitrogen) in two opposing orientations in pHYGGS (Fox et al., 2008), resulting in a final gene silencing vector. The lack of mutations was confirmed by sequencing using primers TGTGTCCATCATGGTGCTGAG and GAAGCCCGACCTCGTTCTG, for the 1st and 2nd insertion, respectively. The final vector was used for *Agrobacterium tumefaciens* (strain LBA4404) -mediated DNA delivery into conidia of the *L. maculans* isolate JN3 according to (Gardiner and Howlett, 2004). Transformants were subjected to three rounds of selection on hygromycin (50 μg ml^-1^). In parallel, a control non-transformed line JN3-WT was subjected to all rounds of the cefotaxime exposure used to clear out Agrobacterium. At least 10 mutant fungal lines were generated and analyzed for each construct, assessing the level of target transcripts by q-PCR, CK spectra, growth and virulence and comparing to the wild-type line. To minimize the unlikely effects of ectopic integrations of T-DNA, analyses were performed with at least three independent lines, including also a line exhibiting a weak level of silencing at the transcriptional level, which served as a negative control.

### Gene Expression Analysis

Total RNA was isolated from 150 mg of frozen fungal mycelium or plant tissue using a Spectrum Plant Total RNA Kit (Sigma–Aldrich, St. Louis, MO, United States) and treated with a DNA-free Kit (Ambion, Austin, TX, United States). Then 1 μg of RNA was used for reverse transcription to cDNA with M-MLV RNase H– Point Mutant reverse transcriptase (Promega Corp., Fitchburg, WI, United States) and anchored oligo dT_21_ primer (Metabion, Martinsried, Germany). Gene expression was quantified by q-PCR using LightCycler 480 SYBR Green I Master kit and LightCycler 480 (Roche, Basel, Switzerland). The PCR conditions were: 95°C for 10 min, followed by 45 cycles of 95°C for 10 s, 55°C for 20 s, and 72°C for 20 s, followed by a melting curve analysis. Relative expression was calculated with efficiency correction and normalization to the housekeeping gene Lm*Tubulin*. Primers were designed using PerlPrimer v1.1.21. A list of the *L. maculans* genes, corresponding accession numbers and primers was as follows: *LmTubulin*, XM_003836006, F: TCAAGATGTCCTCCACCT, R: GTACCAATGCAAGAAAGCC; *LmIPT*, XM_003842009, F: TGACAAGATGTTACAACGAG, R: ATACTGGAGTTTGAGCGA; *LmAK*: XM_003842992, F: GTCAAGCAAGTCCCTGTC, R: ATGTCAATCGCCTTCTCC.

### Analysis of Plant Hormones

For hormone analysis, 150 mg of fresh material from plant tissue pooled out of eight different plants, 25 mg of freeze-dried and homogenized material of fungal mycelium, or 500 μl of cultivation media, was used. Levels of plant hormones were determined as previously described (Dobrev and Kaminek, 2002). Briefly, samples were homogenized in tubes with 1.3 mm silica beads using a FastPrep-24 instrument (MP Biomedicals, CA, United States) with extraction reagent methanol/H_2_O/formic acid (15:4:1, v:v:v) supplemented with stable isotope-labeled CK internal standards, each at 10 pmol per sample, to check the recovery during the purification and to validate the quantification. Clarified supernatants were subjected to solid phase extraction using Oasis MCX cartridges (Waters Co., Milford, MA, United States). The eluates were evaporated to dryness and the generated solids were dissolved in 30 μl of 5% (v/v) methanol in water. Quantification was done on an Ultimate 3000 high-performance liquid chromatograph (HPLC; Dionex, Bannockburn, IL, United States) coupled to a 3200 Q TRAP hybrid triple quadrupole/linear ion trap mass spectrometer (MS; Applied Biosystems, Foster City, CA, United States) as described in (Djilianov et al., 2013). Metabolite levels were expressed in pmol/g dry or fresh weight (DW or FW), for mycelium or plant tissue, respectively, or in pmol/ml of cultivation media. Abbreviations of CK metabolites are adopted and modified according to (Kamínek et al., 2000).

### Measurement of Cytokinin Oxidase/Dehydrogenase Activity

Mycelium of *L. maculans* collected after 7 or 9 days of cultivation in liquid Gamborg medium, frozen in liquid nitrogen and lyophilized, was analyzed for the CKX activity using [2-^3^H]-labeled CKs ([2-^3^H]iP, [2-^3^H]*t*Z and [2-^3^H]*c*Z) as substrates according to (Gajdošová et al., 2011). The *in vitro* assay mixture (50 μl final volume) included 100 mM MES-NaOH or TAPS-NaOH buffer containing 75 μM 2,6-dichloroindophenol (pH 5.7 or pH 8.5, respectively), 2 μM [2-^3^H]CK (7.4 TBq mol^-1^ each) and the mycelium preparation (equivalent to ca. 0.3 mg protein g^-1^ for [2-^3^H]iP or 3 mg protein g^-1^ for [2-^3^H]*t*Z and [2-^3^H]*c*Z as substrates). After incubation (overnight at 37°C), the reaction was terminated by the addition of 95% (v/v) cold ethanol (120 μL) and 200 mM Na_4_EDTA (10 μL). Separation of the substrate from the product of the enzyme reaction was achieved by HPLC as described by (Gaudinová et al., 2005). Protein concentrations were determined according to the method of (Bradford, 1976) using bovine serum albumin as a standard. For each buffer, the analyses of CKX activity were performed in two independent experiments, each assay being done in duplicate. The results are presented as means ± SE.

### Homology and Phylogenetic Analyses

Protein homology searches were performed using BLASTp implemented in NCBI. For *L. maculans*, queries were compared to JN3 genome (Rouxel et al., 2011). Protein sequences were aligned with the MUSCLE program as implemented in www.phylogeny.fr (Dereeper et al., 2008). The Maximum-likelihood phylogenetic tree was generated with SeaView version 4 software (Gouy et al., 2010) using a LG substitution model and bootstrapping with 100 replications. The tree was displayed with MEGA 5.2.2. software (Tamura et al., 2011).

### Statistical Analyses

If not mentioned otherwise, all experiments were repeated three times, with at least two technical replicates. All statistical analyses were performed with Microsoft Excel 2013. The P values were calculated using a two-tailed Student’s *t*-test.

## Results

### Infection with *L. maculans* Alters CK Profile in *B. napus* Cotyledons

As some phytopathogenic microorganisms, including fungi, modify CK levels in host plants (Behr et al., 2012; Jiang et al., 2013; Hinsch et al., 2015; Morrison et al., 2015), we have investigated whether this scenario is also valid for the compatible interaction *L. maculans* (*Lm*) – *B. napus*. Plants of *B. napus* susceptible cultivar Eurol were inoculated by conidia of *L. maculans* isolate JN3 (Balesdent et al., 2001; Rouxel et al., 2011; Šašek et al., 2012b). *L. maculans* grew asymptomatically until 8 days post inoculation (dpi), when grayish lesions appeared. CKs were determined by HPLC-MS analysis at 7 and 10 dpi, corresponding thus to the biotrophic and necrotrophic phase of the *L. maculans* lifestyle *in planta*, respectively. In general, *t*Z and iP-types predominated in the spectrum of CKs in *B. napus*, with *N*7-glucosides, namely *t*Z-*N*7-glucoside (*t*Z7G) and iP-*N*7-glucoside (iP7G), representing the most abundant forms (**Table 1**). At 7 dpi, CK levels remained statistically unaffected by the infection. With the progression of the infection, the levels of most CK forms increased at 10 dpi (**Table 1**). The total CK content increased to 150% compared to mock-infected samples. The highest (240%) increase was observed for *c*Z-type CKs. All of the detected *c*Z-type derivatives were induced by infection at 10 dpi, with the free *c*Z and *c*Z-*N*7-glucoside (*c*Z7G) reaching the highest concentrations (**Table 1**). These data show that infection with *L. maculans* modifies significantly the content of CKs in oilseed rape cotyledon leaves.

**Table 1:** Cytokinin content in *L. maculans*- and mock-infected *B. napus* cotyledons.

	7 dpi			10 dpi		
CK metabolite	Mock	*Lm*-infected	FC (%)	Mock	*Lm*-infected	FC (%)
iP	0.11 ± 0.05	0.50 ± 0.11	435	0.07 ± 0.04	0.25 ± 0.06	350
iP7G	23.62 ± 0.68	26.63 ± 0.81	113	23.45 ± 0.65	34.88 ± 0.80	149^∗^
iP9G	0.14 ± 0.06	0.53 ± 0.04	384^∗^	0.15 ± 0.02	0.31 ± 0.06	198
**Total iP**	**24.40 ± 0.56**	**27.97 ± 0.72**	**115^∗^**	**23.83 ± 0.55**	**35.82 ± 0.87**	**150^∗^**
*t*Z	2.22 ± 1.04	0.73 ± 0.18	33	5.72 ± 1.09	5.63 ± 0.27	98
*t*ZR	0.21 ± 0.11	nd		0.30 ± 0.10	0.77 ± 0.21	253
*t*Z7G	44.01 ± 4.43	39.02 ± 0.92	89	42.12 ± 4.61	57.85 ± 3.60	137
*t*Z9G	7.26 ± 1.26	4.66 ± 0.51	64	5.97 ± 0.90	5.48 ± 0.08	92
*t*Z*O*G	10.92 ± 2.67	5.18 ± 1.93	47	6.41 ± 1.28	10.91 ± 2.40	170
*t*ZRMP	0.14 ± 0.12	0.06 ± 0.05	45	0.26 ± 0.08	1.54 ± 0.49	580
**Total *t*Z**	**65.01 ± 7.85**	**49.66 ± 0.81**	**76**	**60.78 ± 7.90**	**82.40 ± 6.10**	**136**
*c*Z	0.95 ± 0.07	1.49 ± 0.45	157	1.16 ± 0.47	4.36 ± 0.46	375^∗^
*c*ZR	0.11 ± 0.05	0.59 ± 0.18	543	0.08 ± 0.01	0.92 ± 0.27	1178^∗^
*c*Z7G	8.15 ± 0.46	7.76 ± 0.73	95	6.53 ± 0.34	11.11 ± 0.88	170^∗^
*c*ZR*O*G	0.25 ± 0.20	nd		nd	1.56 ± 0.68	
*c*ZRMP	0.14 ± 0.12	0.23 ± 0.02	163	0.08 ± 0.05	0.56 ± 0.03	655^∗^
**Total *c*Z**	**9.60 ± 0.37**	**10.07 ± 1.02**	**105**	**7.85 ± 0.47**	**18.50 ± 1.75**	**236^∗^**
DHZ7G	3.41 ± 0.41	2.71 ± 0.42	80	3.48 ± 0.35	5.68 ± 0.62	163^∗^
**Total DHZ**	**3.89 ± 0.40**	**3.28 ± 0.27**	**84**	**4.06 ± 0.50**	**6.42 ± 0.53**	**158^∗^**
**Total CKs**	**102.89 ± 8.54**	**90.98 ± 0.73**		**96.53 ± 8.27**	**143.14 ± 6.78**	**148^∗^**

*B. napus cotyledons cv. Eurol were inoculated by *L. maculans* (*Lm*) isolate JN3. Four biological replicates were analyzed at two time points (7 and 10 days post inoculation, dpi). Data represent mean concentrations (pmol CK g^-*1*^ fresh weight, FW) ± SE from one experiment of two. Only metabolites where at least one concentration value is higher than 0.50 are shown. nd, not detectable; differences between *Lm*-infected and mock-infected cotyledons are expressed as a fold change (FC) and those significant are marked (^∗^*P* < 0.05). Total sums of CKs of different types are highlighted in bold.*

### *L. maculans* Produces a Wide Range of CKs

**FIGURE 1:**
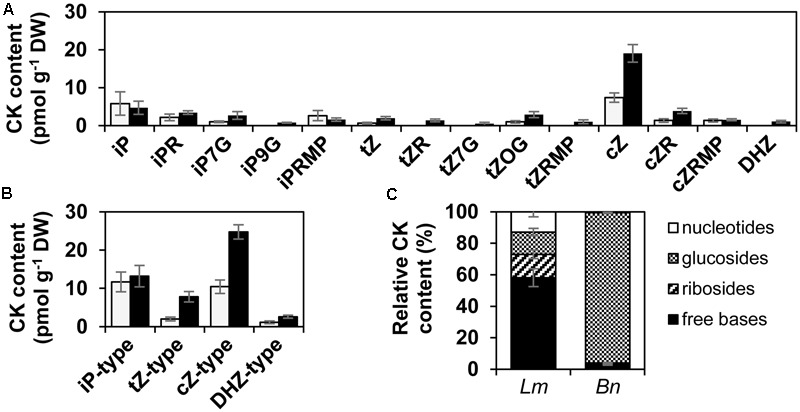
Cytokinin content in *L. maculans* mycelium grown *in vitro*. **(A,B)** Representative profile of endogenous CKs in *L. maculans* mycelium grown *in vitro* at 7 days (gray bars) and 9 days (black bars). **(A)** Spectrum of distinct CK derivatives displaying those with higher concentration than 0.5 pmol.g^-1^ dry weight (DW). **(B)** The sum of all iP-, *t*Z-, *c*Z and DHZ-type CKs. **(C)** Relative distribution of CK groups in 7-day-old mycelium (*Lm*) compared to CK distribution in *B. napus* (*Bn*) non-infected cotyledons. All data represent mean values ± SE (pmol CK g^-1^ dry weight, DW) out of three experiments.

To assess the possibility that the induction of CKs in oilseed rape cotyledons might be of fungal origin, the ability of the hemibiotrophic fungus *L. maculans* to produce CKs was assessed *in vitro*. Mycelia of the isolate JN3 were grown for 7 and 9 days in a liquid culture in Gamborg medium and analyzed for the presence of CKs by liquid chromatography coupled to mass spectrometry. At both mycelial ages, the *c*Z- and iP-type CKs predominated, followed by *t*Z-type derivatives, while DHZ occurred in considerably lower amounts and in a less reproducible manner (**Figures 1A,B**). All CK types were mainly present as free bases (**Figures 1A,C**). As shown for the 7-day old mycelium, free bases, and free bases together with their ribosides, represented 58 and 75% of all detected CK derivatives, respectively (**Figure 1C**). Other CK derivatives, i.e., glucosides or nucleotides, were less abundant. Among the glucosides, *O*-glucosides of *t*Z (*t*Z*O*G) and *N*7-glucosides (iP7G, *t*Z7G) were detected, whereas *N*9-glucosides were mostly missing in the mycelium (**Figure 1A**). The most abundant metabolite of the *t*Z-type CKs was *t*Z*O*G. Also, riboside monophosphates were detected at both mycelial stages, although their levels were relatively low (**Figures 1A,C**). At 9 days, the sum of total CKs in the mycelium increased compared to 7 days, mainly due to the increase of *c*Z-type CKs, the free *c*Z especially being the highly predominant CK derivative (**Figure 1B**). To sum up, *L. maculans* can produce CKs *in vitro* and its CK profile differs from that of the host *B. napus* tissue (**Figure 1C**).

### *L. maculans* Metabolizes Exogenously Added CKs

**FIGURE 2:**
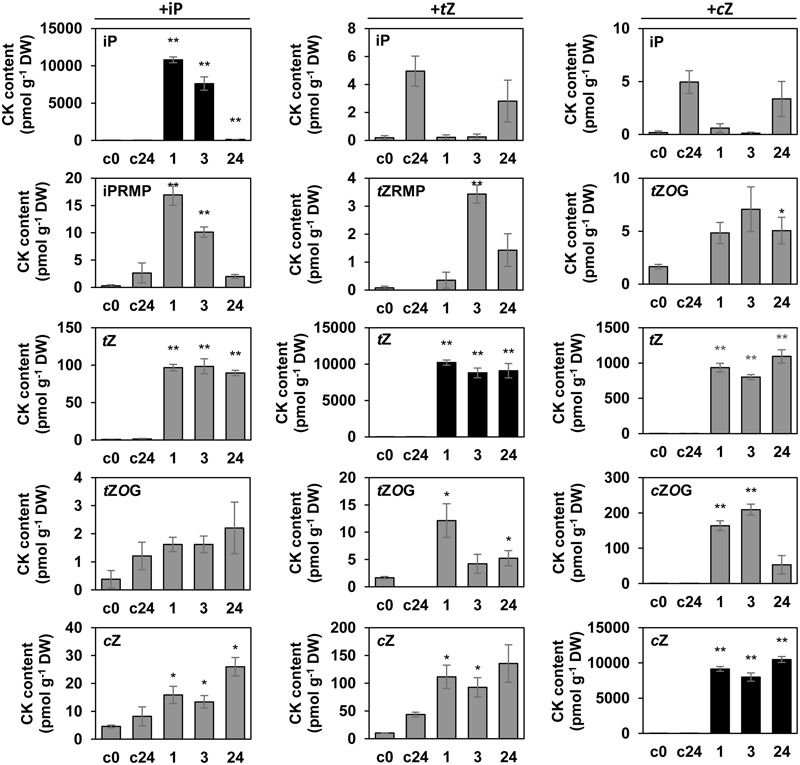
*In vitro* conversion of individual cytokinins by *L. maculans*. 7-day old mycelium of *L. maculans* cultivated *in vitro* was treated with 1 μM CK (iP, *t*Z, *c*Z) for 1, 3, and 24 h. The content of endogenous CKs in mycelium was compared to control samples c0 (non-treated control at time 0) and c24 (mock-treated for 24 h). Data represent mean values (pmol CK g^-1^ DW) ± SE of biological triplicates, repeated twice. The particular CK added is mentioned above each column of graphic, detected CK is mentioned within each graphic. Black bars represent the detection of CK metabolite added. Asterisks indicate significant differences between the CK-treated sample and the corresponding control (^∗^*P* < 0.05, ^∗∗^*P* < 0.01). Except from all three CK bases, only CK metabolites showing alterations are displayed.

By showing that *L. maculans* produces a wide range of CKs *in vitro* and possibly *in vivo*, we aimed to get some insight into the CK metabolic and biosynthetic pathways. Two approaches were undertaken: CK feeding assays; and a silencing strategy targeting the genes involved in CK metabolism. First, the ability of *L. maculans* to convert exogenously added CKs was assessed. Mycelium was grown in a liquid culture for 7 days, treated with exogenous solutions of iP, *t*Z, *c*Z (all at a final concentration of 1 μM) or treated with mock. CK content was then analyzed at 0, 1, 3, and 24 h and compared to the corresponding mock-treated sample. For the 1 and 3 h time points, CK-treated samples were compared to time 0 (c0), as the mock treatment did not affect the CK levels at these early time points (data not shown). The measured levels of individual CKs added to the medium reached around 10,000 pmol.g^-1^ of dry weight (DW) in the mycelium from the very 1st hour (**Figure 2**). The levels of endogenous iP after iP-feeding degraded progressively with time, with less than 150 pmol.g^-1^ DW remaining after 24 h, while the levels of both zeatin bases were stable over time and a visible decrease did not occur. Detected metabolites issued from the CK conversions were mainly the free CK bases, *O*-glucosides and riboside monophosphates (**Figure 2**), whereas only minor amounts of ribosides were formed. Following feeding with iP, iP-9-riboside-5′-monophosphate (iPRMP) was the main metabolite observed in *L. maculans*. The conversion of iP to iPRMP was rapid, with the iPRMP peaking at 1 h post treatment, and a subsequent gradual decrease of its concentration to a barely detectable value at 24 h post treatment, in comparison to the mock treatment. Evidently, iP was substantially converted to the free *t*Z base, which increased up to 100 pmol.g^-1^ DW, representing thus a 100-fold increase, within the 1st hour. Following the next 24 h, the level of *t*Z in *L. maculans* mycelium remained unchanged, whereas a slight increase in *t*Z*O*G content was observed, reaching its maximum at 24 h. The concentration of *c*Z also gradually increased, though reaching lower levels than *t*Z. Feeding with *t*Z also led to a transient increase in *t*Z*O*G and further in *t*Z-9-riboside-5′-monophosphate (*t*ZRMP), with maximum values at 1 and 3 h, respectively. The *t*Z feeding also increased *c*Z amounts up to 110 pmol.g^-1^ DW from the 1st hour with no further changes over time. The *c*Z was metabolized into *c*Z*O*G, with its concentration reaching about 200 pmol.g^-1^ DW at 3 h. Interestingly, feeding with *c*Z led to a massive increase in the *t*Z level (up to 1000 pmol.g^-1^ DW), which suggests a process of *cis*–*trans* isomerisation in *L. maculans*. Also detected following *c*Z feeding was *t*Z*O*G. No iP derivatives were detected after either *t*Z or *c*Z feeding (**Figure 2**). A parallel analysis of CKs in the *L. maculans* cultivation medium before, and 24 h after, the adding of individual CKs (Supplementary Figure 1) showed increased contents of free bases and *O*-glucosides, in agreement with the analyses from the mycelia, but with the exception of riboside monophosphates that were not detected in the medium.

### Zeatin *cis*–*trans* Isomerisation in *L. maculans*

**FIGURE 3:**
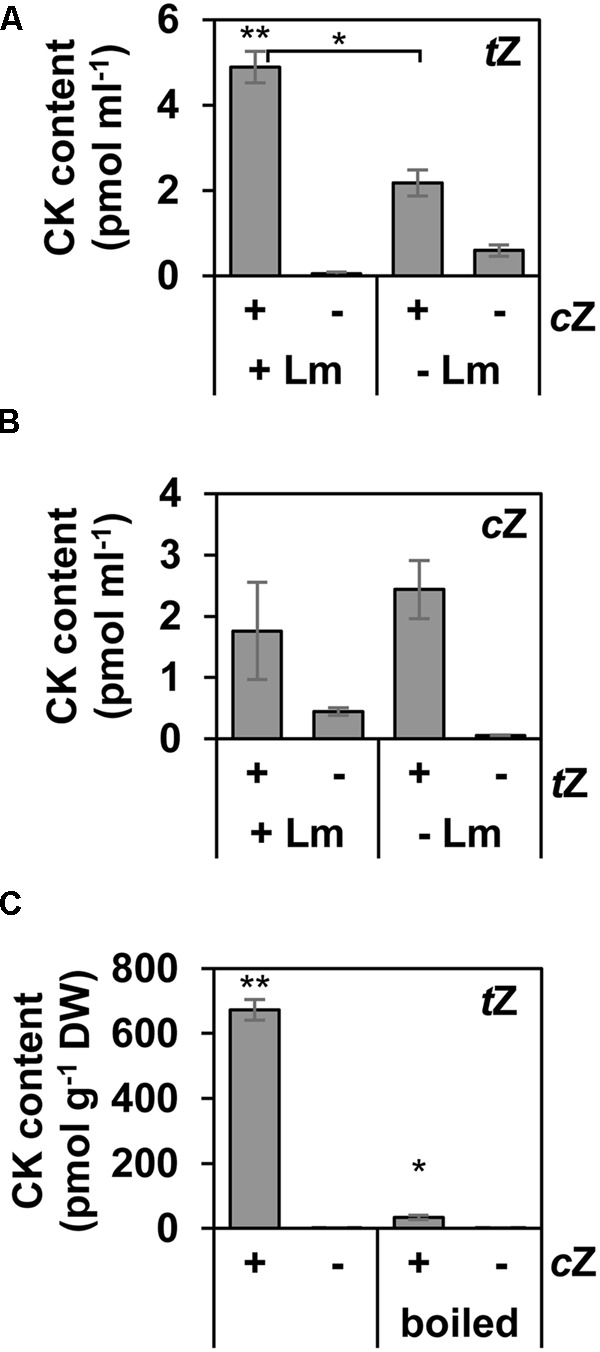
Zeatin *cis*–*trans* isomerisation activity in *L. maculans in vitro*. Content of *t*Z **(A)** and *c*Z **(B)** in the cultivation medium with (+Lm) or without (–Lm) the *L. maculans* mycelium 24 h after feeding with the other zeatin isomer. **(C)** Content of *t*Z in the mycelium, boiled or not, 3 h after adding *c*Z. CK treatment (+) is indicated outside the right bottom corner of each graphic and compared with the control mock treatment (–). The detected CK is mentioned in the right upper corner of each graphic. Unless otherwise indicated, asterisks indicate significant differences between the CK- and the corresponding mock-treated variant (^∗^*P* < 0.05, ^∗∗^*P* < 0.01). Data represent mean values ± SE of biological triplicates, repeated twice. For **(C)**, other altered CK derivatives are shown in Supplementary Figure 2.

The previous data suggested that *L. maculans* may exhibit zeatin *cis*–*trans* isomerase activity. To also consider the possible non-enzymatic source of *t*Z, controls were carried out, where (i) CK was added to the cultivation medium not inoculated with *L. maculans*; and (ii) CK was added to a *L. maculans* culture killed by boiling. Concerning the generation of *t*Z from *c*Z, we show that some amount of *t*Z accumulates spontaneously in the medium 24 h after *c*Z feeding, even in the absence of fungus. However, the *t*Z amount was significantly higher in the presence of the fungus than in its absence, indicating that the *cis*–*trans* isomerase activity in *L. maculans* really does occur (**Figure 3A**). On the other hand, the amount of *c*Z formed from exogenously added *t*Z in the medium did not depend on the presence of *L. maculans* - bringing no clear evidence on the *trans*–*cis* isomerisation in the fungus (**Figure 3B**). Besides, when *c*Z was added to the previously boiled culture, *t*Z formation was compromised: *t*Z reaching about 20-times lower concentrations than in the non-boiled culture (**Figure 3C**). Furthermore, the minor formation of *t*Z*O*G was absent in the boiled culture (Supplementary Figure 2). Conversion into other *c*Z-derived metabolites, such as *c*Z*O*G and *c*ZRMP, was also compromised in the boiled culture (Supplementary Figure 2), suggesting an active *L. maculans* metabolism involved in the formation of CK glucosides and riboside monophosphates.

### *L. maculans* Can Degrade iP via Its CKX Activity

**FIGURE 4:**
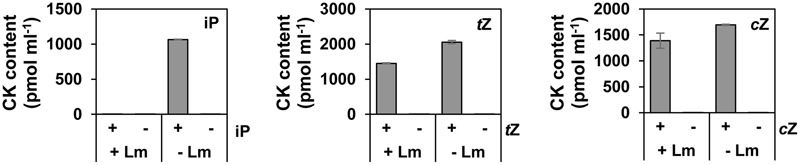
Cytokinin degradation by *L. maculans*. Content of endogenous CK base in the cultivation medium with (+*Lm*) or without fungus (–*Lm*) after 24 h *post* treatment with exogenous CK (iP, *t*Z, or *c*Z) at 1 μM concentration (+) or mock (–). Data represent mean values (pmol CK ml^-1^ of medium) ± SE of biological triplicates.

**FIGURE 5:**
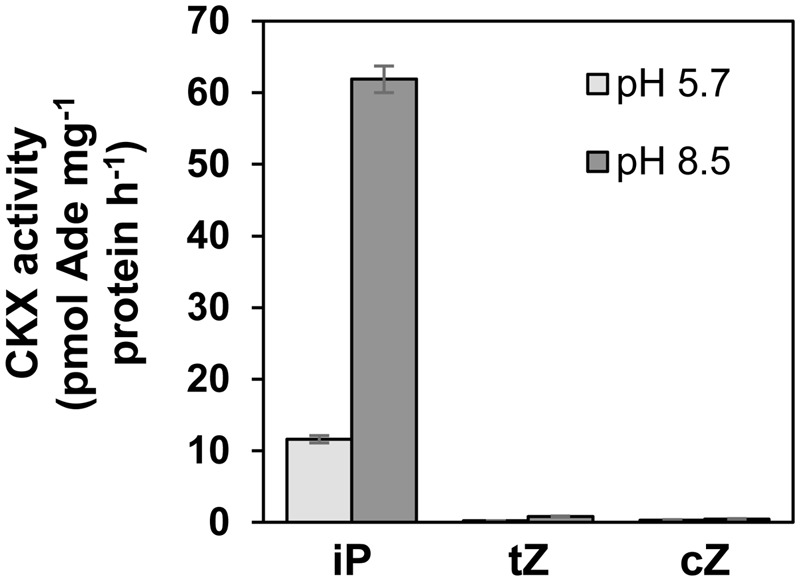
Cytokinin oxidase/dehydrogenase (CKX) activity of *L. maculans*. The enzyme assay was carried out *in vitro* using the protein preparation of 7-day-old mycelium, incubated with [2-^3^H]-labeled CK substrates (iP, *t*Z, or *c*Z) in MES-NaOH (pH 5.7) or TAPS-NaOH (pH 8.5) buffers. The CKX activity was assessed as the amount of [2-^3^H]-adenine (Ade) formed in time related to the protein mass (in pmol Ade mg^-1^ protein h^-1^). Data represent mean values ± SE of two biological experiments, each in duplicates.

Our data show that after a 24-h period only a limited amount of exogenously added iP remained in the mycelium (**Figure 2**) and lower than 0.1 pmol ml^-1^ concentrations were detectable in the cultivation medium (**Figure 4**). In contrast to that of iP, both zeatin bases remained stable over time, both in the mycelium and the medium (**Figures 2, 4**). A degradation of iP was not detected in the Gamborg medium itself in the absence of the fungus and thus degradation fully depended on the presence of *L. maculans* (**Figure 4**). Moreover, no increase in known iP-derived metabolites, such as ribosides, nucleotides, or glucosides, which could explain the massive decrease in iP concentration, was observed either in the mycelium or the medium. Therefore, the possibility of CK degradation was then investigated. In plants, CKs are mainly degraded by CKX activity (Sakakibara, 2006). However, there are no data yet available on the CKX activity in fungi. The CKX activity of *L. maculans* mycelium was thus assessed in the protein extract of 7 day-old mycelium cultivated *in vitro* with [2-^3^H]-labeled CKs (**Figure 5**). Interestingly, [2-^3^H]iP was found to be degraded: the detected CKX enzyme activity reaching 12 pmol adenine (Ade) mg^-1^protein h^-1^ in the MES-NaOH buffer (pH 5.7) and 64 pmol Ade mg^-1^ protein h^-1^ in the TAPS-NaOH buffer (pH 8.5). Therefore, a higher CKX activity was detected in the more alkaline pH. In contrast, the CKX activity with the use of labeled zeatins, [2-^3^H]*t*Z and [2-^3^H]*c*Z, was only minute, showing values below 1 pmol Ade mg^-1^ protein h^-1^. The CKX activity was also measured in 9-day old mycelium giving very similar results (data not shown). Our data clearly demonstrate significant CKX activity for iP, while being very low for *t*Z and *c*Z, in *L. maculans*.

### The Effect of CKs on *L. maculans* Growth *In Vitro*

Given the differences in metabolism of iP, *t*Z, and *c*Z, one can suggest that particular CKs might play different biological roles in the *L. maculans* mycelium. Therefore the effect of iP, *t*Z, and *c*Z on *L. maculans* growth *in vitro* was investigated. For the growth assays, conidia of *L. maculans* expressing sGFP (JN3-GFP) were germinated in Gamborg medium supplemented with these CKs and CKs *N*^6^–benzyladenine (BA) and kinetin (KIN) for comparison. The concentration range used was 2 nM to 100 μM (iP, *t*Z, and *c*Z) or 1 mM (BA and KIN). Fluorescence demonstrating mycelial growth was measured from 3 to 7 days and compared to mock DMSO-treated samples. No effect of iP, *t*Z, or *c*Z on the GFP fluorescence reflecting growth was observed for any concentration and time period tested. Data for the treatment with isoprenoid CKs at the highest concentration 100 μM are shown (Supplementary Figure 3A). On the other hand, treatment with BA and KIN induced growth inhibition at concentrations 63 μM – 1 mM (Supplementary Figure 3B). To sum up, besides the inhibition by BA and KIN, neither growth-promoting, nor inhibitory effects of iP, *t*Z, or *c*Z were observed and thus the biological roles of these CKs could not be judged by means of this analysis.

### Identification of CK Biosynthetic and Metabolism Genes in *L. maculans*

The second approach to unravel the CK metabolic pathways in *L. maculans* consisted in the silencing of key CK enzymes. Specifically, we were interested in two enzymes, the biosynthetic enzyme IPT, and the AK involved in the recycling of free CK bases into their corresponding nucleotides in plants. Orthologs of these plant enzymes were identified in *L. maculans* and functional characterization was performed using post-transcriptional silencing by RNAi. In the generated mutant fungal lines, CKs were determined in mycelia grown in liquid cultures *in vitro* for 9 days (when their levels reached higher values than at 7 days; **Figures 1A,B**).

### Isopentenyl Transferase of *L. maculans* Is Involved in the Production of *c*Z

**FIGURE 6:**
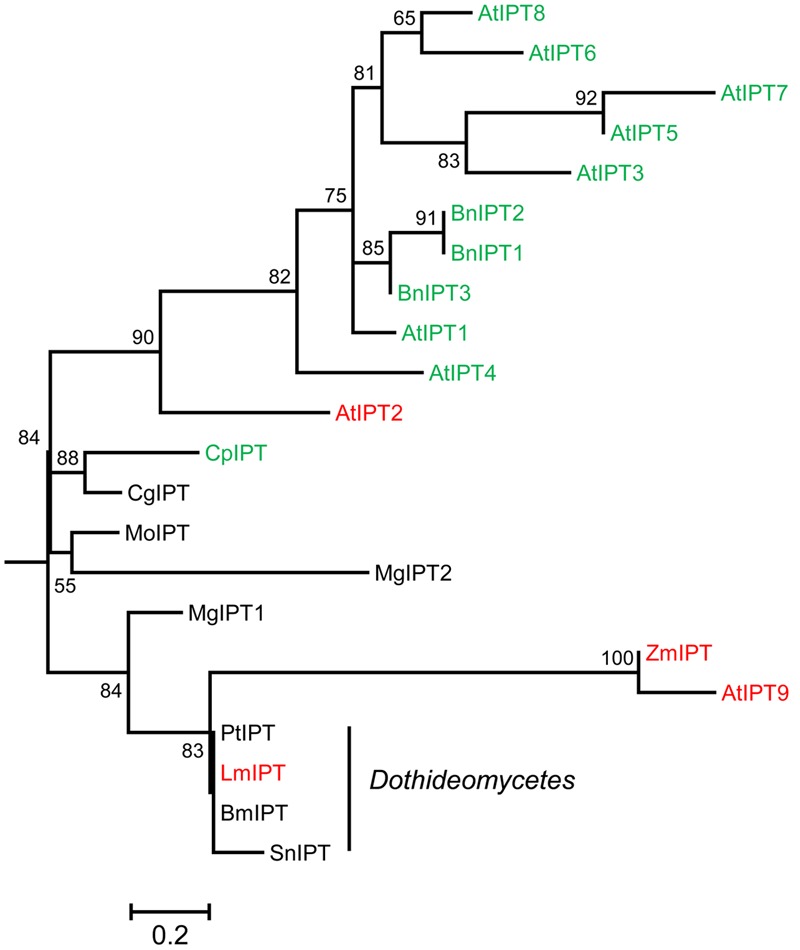
Isopentenyltransferase phylogeny. Maximum-likelihood phylogenetic tree showing the relationship between the protein sequences (GenBank) of the isopentenyltransferases (IPTs) of Arabidopsis (AtIPTs), *Brassica napus* (BnIPTs), the homolog from *L. maculans* (LmIPT) and other fungi. Only bootstraps higher than 50 (from 100) are presented. Sequences are highlighted in color according to the IPT type: green, adenylate-IPTs; red, tRNA-IPTs. *Zm, Zea mays*; *Mo, Magnaporthe oryzae*; *Mg, Mycosphaerella graminicola*; *Pt, Pyrenophora tritici-repentis*; *Bm, Bipolaris maydis*; *Sn, Stagonospora nodorum*; *Cg, Colletotrichum graminicola; Cp, Claviceps purpurea.*

**FIGURE 7:**
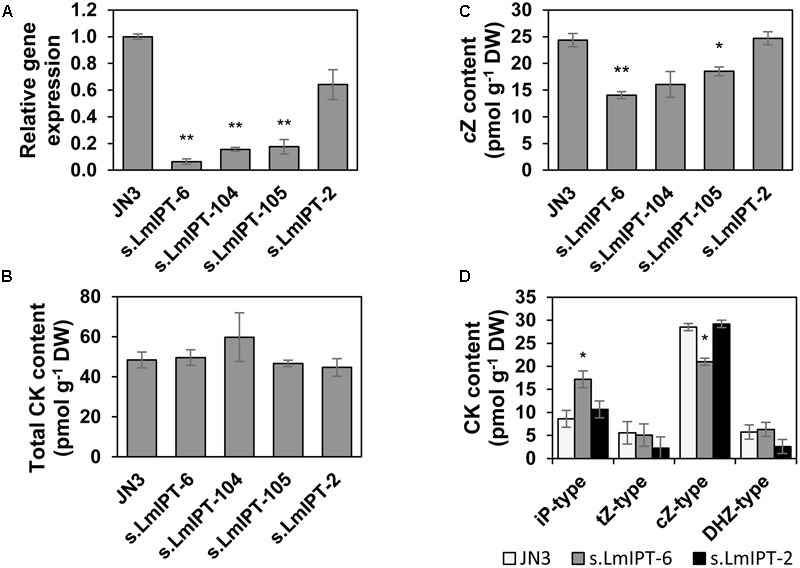
Characterization of LmIPT silenced mutants of *L. maculans*. Wild-type *L. maculans* (JN3) and different silenced LmIPT (s.LmIPT) transformants were cultivated in a liquid culture *in vitro* and mycelium was sampled at 9 days. **(A)** Expression of *LmIPT* gene was analyzed by qPCR, normalized to *LmTUB* and reported to WT. **(B–D)** Endogenous CK content in mycelium expressed as pmol g^-1^ DW ± SE: **(B)** *c*Z content, **(C)** the total CK content and **(D)** the sum of all iP-, *t*Z-, *c*Z and DHZ-type CKs in s.LmIPT-6 and s.LmIPT-2 lines. Data are from three biological experiments for each assay. Asterisks indicate significant differences between transformants and JN3 (^∗^*P* < 0.05, ^∗∗^*P* < 0.01).

In plants and bacteria, CKs are biosynthesized by IPTs and recent studies have shown that fungi use similar enzymes (Hinsch et al., 2015; Chanclud et al., 2016). Using the AtIPT1-AtIPT9 from *Arabidopsis thaliana* as a query, only one predicted protein was detected in *L. maculans*: XP_003842057, further designated as LmIPT. The putative gene *LmIPT* (1525pb; 2 introns) encodes a protein of 475 amino acids (aa). LmIPT contains a predicted conserved tRNA Δ^2^-isopentenylpyrophosphate transferase domain (PRK00091) present in the *miaA* gene product of *Escherichia coli* (Connolly and Winkler, 1991). Based on a phylogenetic analysis, LmIPT share a high homology with AtIPT9 (*E* = 5.0–50; 93% query cover; 31% identity), characterized as tRNA-IPT (Miyawaki et al., 2006; **Figure 6**). Furthermore, other *Dothideomycete* fungi genomes carry only one ortholog exhibiting high similarity to AtIPT9, which leads to *c*Z biosynthesis in Arabidopsis. Analyses based on q-PCR in selected lines silenced in LmIPT (s.LmIPT lines) showed a decrease in *LmIPT* transcripts below 20% in lines s.LmIPT-6, -104, and -105 (**Figure 7A**). In these silenced lines, the level of *c*Z was significantly decreased (**Figure 7B**) and well correlated with the extent of *LmIPT* transcript silencing. In the best silenced line s.LmIPT-6, the level of *c*Z significantly decreased (to 57% compared to the wild-type control line). In contrast, the line s.LmIPT-2, with a residual *LmIPT* transcript level reaching about 60%, did not exhibit any alteration in *c*Z levels. Besides the decrease in *c*Z levels, no other CK metabolites were significantly declined in s.LmIPT transformants. Interestingly, the total CK levels were not altered in s.LmIPT fungi (**Figure 7C**) since iP-type derivatives (such as iP) were increased (**Figure 7D**). To sum up, these data indicate that LmIPT is involved in *c*Z production.

The *in vitro* growth of s.LmIPT transformants was also assessed. No difference in the radial growth was observed on solid V8 agar plates (Supplementary Figure 4A) and only a slightly decreased mycelial yield was recorded in liquid cultures compared to the JN3 wild type (Supplementary Figure 4B), though not correlated with *LmIPT* transcript levels (**Figure 7A**). No aberration in hyphal morphology was observed: either on the V8 plates or in the liquid culture in the Gamborg medium (data not shown). In addition, the virulence of s.LmIPT transformants was assessed on *B. napus* cotyledons. The area of grayish lesions formed at 10 dpi relative to the leaf surface was measured to evaluate disease severity. No differences in the area of leaf necrosis were found for s.LmIPT transformants compared to the JN3 infection (Supplementary Figure 4C). Overall, no considerable impact of LmIPT silencing on fungal morphology, growth and virulence was observed.

### Adenosine Kinase Converts CK Ribosides into CK Monophosphates

**FIGURE 8:**
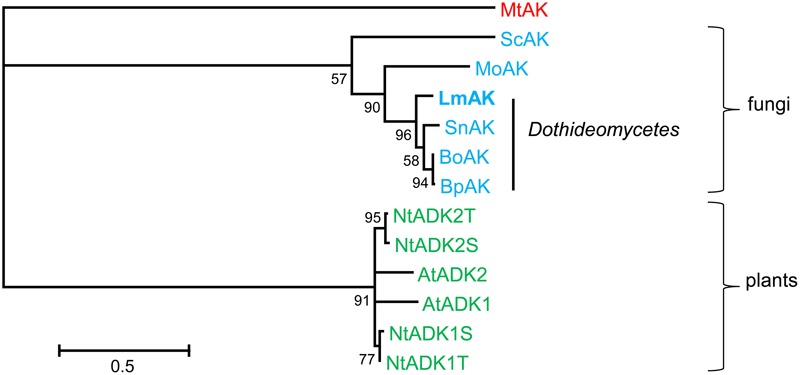
Adenosine kinase phylogeny. Maximum-likelihood phylogenetic tree showing the relationship between the protein sequences (GenBank) of the adenosine kinases (AK) of Arabidopsis (AtADKs), *Nicotiana tabacum* (NtADKs), the homolog from *L. maculans* (LmAK) and other fungi. The sequence of *Mycobacterium tuberculosis* (*Mt*) was used as the outgroup. Only bootstraps higher than 50 (from 100) are presented. Sequences are highlighted in color according to the species origin: green, plant; blue, fungi; red, bacteria. *Mo, Magnaporthe oryzae*; *Sc, Schizophyllum commune*; *Bp, Bipolaris maydis; Bo, Bipolaris oryzae; Sn, Stagonospora nodorum.*

**FIGURE 9:**
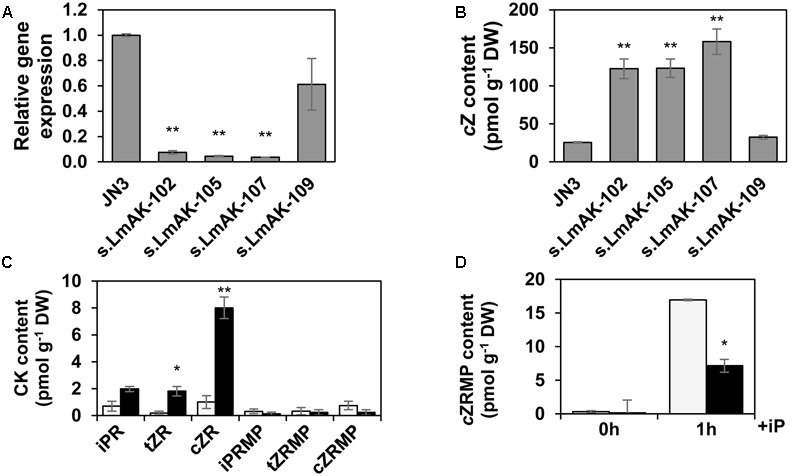
Characterization of LmAK silenced mutants of *L. maculans*. **(A,B)** Wild-type *L. maculans* (JN3) and different silenced LmAK (s.LmAK) transformants were cultivated in a liquid culture *in vitro* and mycelium was sampled at 9 days. **(A)** Expression of *LmAK* gene was analyzed by qPCR, normalized to *LmTUB* and reported to WT. **(B)** *c*Z content in mycelium expressed as pmol g^-1^ DW ± SE. **(C,D)** Content of CK ribosides and monophosphates in wild-type (gray bars) and s.LmAK-105 transformant (black bars) in **(C)** non-treated cultures or **(D)** after treatment with iP (1 μM). Means ± SE from three independent experiments are shown for each assay. Asterisks indicate significant differences between transformants and JN3 (^∗^*P* < 0.05, ^∗∗^*P* < 0.01).

An ortholog to Arabidopsis AK was searched for in *L. maculans* using AtADK1 of Arabidopsis (Moffatt et al., 2000) as a query. *L. maculans* carries only one highly conserved predicted protein, XP_003843040 (*E* = 5.0–105; 95% query cover; 47% identity), further referred to as LmAK. *LmAK* (1248pb; 3 introns) encodes a predicted protein of 349 aa with an annotated AK domain (cd01168) with a conserved substrate and ATP binding sites. Bioinformatic and phylogenetic analyses showed that LmAK shares an extremely high similarity (84–86%) with putative orthologs from other fungi belonging to the same order *Pleosporales* of the *Dothideomycetes* class (**Figure 8**). AK is involved in the recycling of biologically active CKs to restore the CK nucleotide pool (Schoor et al., 2011) by catalyzing the phosphorylation of CK ribosides into monophosphates. Silencing of the *LmAK* gene was successful in generating transformants with residual transcript levels of *LmAK* below 10% compared to the wild type as demonstrated for lines s.LmAK-102, -105, and -107 (**Figure 9A**). Determination of hormone levels in selected mutants grown *in vitro* in liquid Gamborg medium revealed that these three silenced lines exhibited a dramatic fivefold increase in free *c*Z base levels (**Figure 9B**), which impacted the total CK content in all of them (Supplementary Figure 5A). The increase of *c*Z correlated well with the extent of silencing, as another line s.LmAK-109 exhibiting low silencing was not altered in the levels of *c*Z nor in total CK content (**Figure 9B** and Supplementary Figure 5A). The level of *LmIPT* expression was unchanged in s.LmAK lines, so the increase in *c*Z concentration was not due to increased *LmIPT* expression (Supplementary Figure 5B). If AK is involved in the CK metabolism of *L. maculans* and functions analogously as in plants, the silencing of *LmAK* in a fungus should also affect the conversion of CK ribosides into CK monophosphates. As shown for line s.LmAK-105, the levels of ribosides of all three main CKs, i.e., iP, *t*Z, and *c*Z, were increased (**Figure 9C**), being statistically significant for *c*ZR and *t*ZR. The observed alterations were most important for *c*Z-type derivatives. The increase in CK ribosides correlated well with the decline in concentrations of CK riboside monophosphates in s.LmAK-105 (**Figure 9C**). Similar results were also observed for the lines s.LmAK-102 and -107. Nevertheless, as the basal levels of riboside monophosphates are generally low in *L. maculans* mycelium, this difference was not significant. We have shown that *L. maculans* converts exogenously applied iP into iPRMP as a main metabolite (**Figure 2**). Therefore the role of LmAK in iP-induced iPRMP formation was investigated in the line s.LmAK-105. One hour after feeding with exogenous iP, the mycelium of s.LmAK-105 line contained less than 50% of iPRMP compared to the JN3 wild-type (**Figure 9D**). Details on the contents of other CK metabolites in s.*LmAK*-105, including the percentage of alteration, are given in Supplementary Table 1. Furthermore, levels of adenine were increased in line AK-105 compared to the control line (Supplementary Table 1). In summary, our data show that LmAK is: involved in the interconversion of CK ribosides into nucleotides; impacts the accumulation of free bases, *c*Z in particular; and thus contributes to CK homeostasis in the mycelium of *L. maculans*.

### LmAK Is Involved in Fungal Growth and Virulence

**FIGURE 10:**
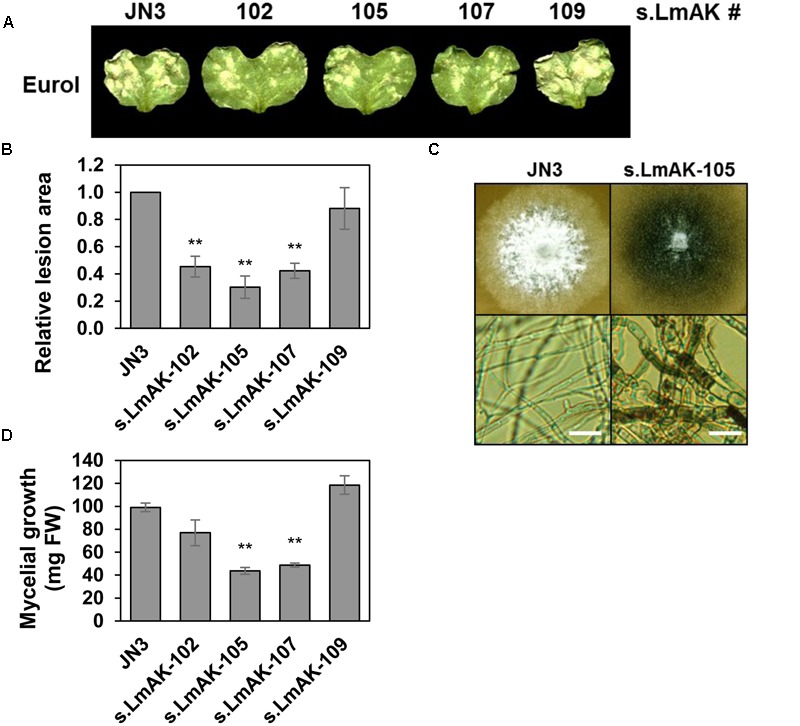
LmAK is involved in fungal virulence and growth. **(A,B)** Infection of JN3 and s.LmAK mutants on cotyledons of *B. napus* cv. Eurol 9 days post inoculation (dpi). **(A)** The panel shows representative infected cotyledons. **(B)** Fungal virulence evaluated as relative lesion area on cotyledons relative to JN3 isolate. Asterisks indicate significant differences between transformants and JN3 (^∗^*P* < 0.05, ^∗∗^*P* < 0.01). **(C)** Macroscopic (up) and microscopic (bottom) phenotype of the 9-day old fungal colony grown on V8 agar plates. Scale bars = 10 μm. **(D)** Growth on V8 plates assessed at 9 days as fresh mycelial weight (FW). All data are means ± SE from at least three independent experiments.

Virulence of s.LmAK transformant lines was investigated on *B. napus* cotyledons. Silenced transformant lines s.LmAK-102, -105, and -107 exhibited a marked decrease in the extent of necrotic lesions compared to wild-type JN3 (**Figures 10A,B**). Silencing of *LmAK* also impacted the growth *in vitro*. While the silenced s.LmAK mutant lines did not exhibit altered morphology or growth rate in liquid Gamborg medium (Supplementary Figure 6A), a marked phenotype was observed when *L. maculans* grew on solid V8 agar medium *in vitro* (**Figures 10C,D**). At 10 dpi of the culture, the mycelium of silenced s.LmAK mutants was sparse and grayish compared to the control mycelium or that of the s.LmAK-109 and -110 lines, not silenced in *LmAK* gene, that remained white and dense (Supplementary Figure 6B). Microscopic analyses of the grayish part of the mycelium from the mutant revealed compact hyphal segments bordered with thick dark-colored cell walls that were missing in the wild-type mycelium (**Figure 10C**). This discernible morphological phenotype became apparent as early as around 5 days of mycelial growth after subculturing on new solid media and correlated well with the altered growth rate. The mass yields of s.LmAK mutants were lower than that of wild-type mycelium (**Figure 10D**).

**FIGURE 11:**
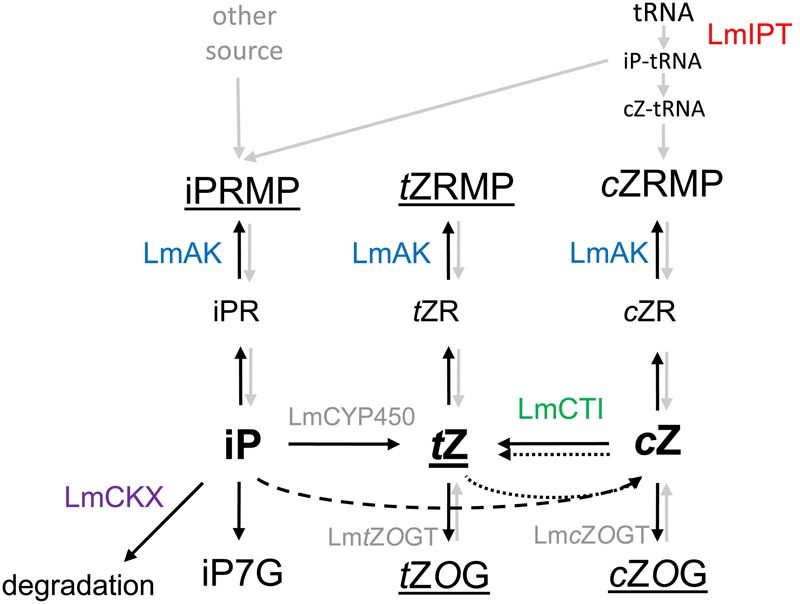
Proposed model illustrating CK metabolism in *L. maculans*. In the model of CK metabolism in *L. maculans*, metabolic pathways supported by the experimental data (black lines), reactions proceeding at lower intensity (dashed black lines) as well as hypothesized and uncharacterized additional conversions (gray lines) are shown. Isopentenyltransferase (LmIPT), an ortholog of Arabidopsis tRNA-IPTs, catalyzes isopentenylation of tRNA. The release of nucleotides from tRNA gives rise to *c*ZRMP and further to *c*Z. Free CK bases are rapidly converted to metabolites (underlined) such as iPRMP and *t*Z (for iP), *t*ZRMP and *t*ZOG (for *t*Z), and *t*Z and *c*ZOG (for *c*Z), therefore a presence of zeatin *O*-glucosyltransferase(s) (*t*ZOG and *c*ZOG, or a non-specific *O*-glucosyltransferase) is envisaged. The iP-CKs are hydroxylated to the *t*Z-CKs, possibly by a cytochrome P450 ortholog (LmCYP450) at the free base level. *c*Z can be enzymatically converted into *t*Z by *cis*–*trans* isomerase (LmCTI). Besides, non-enzymatic interconversions of *c*Z to *t*Z occur in both directions (dotted lines). Adenosine kinase (LmAK) catalyzes phosphorylation of CK ribosides and thus contributes to metabolic pool of CK riboside monophosphates. iP, but not zeatins, can be degraded via the cytokinin oxidase/dehydrogenase (LmCKX) activity. How iPRMP and *t*ZRMP are formed in *L. maculans* remains unclear. Also other steps of CK biosynthesis (gray lines) remain to be characterized including CK nucleotide dephosphorylation and hydrolysis of ribosides to form free CK bases.

In summary, we show that *L. maculans*, a pathogen of oilseed rape, is able to produce, and interconverts, CKs *in vitro*, and alters the CK profile in *B. napus* cotyledons upon infection. Furthermore, the roles of LmIPT, LmAK, LmCKX, and *cis–trans* isomerase in CK metabolism in *L. maculans* have been demonstrated. Based on these findings, we propose a comprehensive model illustrating metabolic conversions in *L. maculans* (**Figure 11**).

## Discussion

### CK Production and Interconversion in *L. maculans*

Different fungi, both pathogenic or symbiotic, have been reported to produce CKs (Crafts and Miller, 1974; Murphy et al., 1997; Jameson, 2000; Chanclud and Morel, 2016). We have shown that *L. maculans* produces a wide spectrum of CKs *in vitro*, present both in the mycelium (**Figure 1**) and culture medium (Supplementary Figure 1), as well as *in planta* upon infection in *B. napus* (**Table 1**). *L. maculans* synthesizes CKs without supplying the cultivation medium with biosynthetic precursors. Furthermore, other phytopathogenic fungi have been reported to produce CKs, such as *M. oryzae* or *C. purpurea* (Jiang et al., 2013; Hinsch et al., 2015; Chanclud et al., 2016). *C. graminicola* synthesizes CKs *in vitro* after feeding with the precursors DMAPP, AMP, ADP, and ATP (Behr et al., 2012). In *L. maculans* mycelium, the CKs of *c*Z-type are dominant with iP-type CKs being the second most abundant (**Figures 1A,B**). Further, *c*Z forms are also the major CKs of *M. oryzae* (Chanclud et al., 2016), and have been proposed as being the main form produced by filamentous fungi (Chanclud and Morel, 2016). It is noteworthy that the profile of produced CKs may reflect the cultivation medium used, as has been reported for *C. purpurea* (Hinsch et al., 2015), or *P. brassicae* (Murphy et al., 1997). In contrast to other fungi studied for their CK production *in vitro*, the CKs of *L. maculans* occurred mostly as free bases, which represented 60% of all detected CK derivatives. In *B. napus*, more than 90% of all CKs are glucosides, where free bases only account for 3% (**Figure 1C**). Therefore, *L. maculans* hyphae behave as a localized source of free CK bases *in planta* and might contribute to the infection process. In dicotyledonous plants, the main CKs are of the iP- or *t*Z-type, as was also observed in *B. napus* cotyledons (**Table 1**). We show here that in the later stages of infection the levels of nearly all *c*Z-type derivatives are increased in *B. napus* cotyledons, which correlates with the fact that *c*Z-type CKs were the main CKs of *L. maculans*, and suggests an origin from the infecting fungus. Considering the increased level of *c*Z7G and its absence in *L. maculans*, it is probable that *c*Z released from *L. maculans* is being metabolized by *B. napus* to biologically inactive form. The induction of iP7G, *t*Z7G and *t*Z*O*G metabolites upon infection suggests of further responses of the plant to the infection. The formation of glucosides might be an efficient plant strategy to clear biologically active CK free bases supplied by the fungus and keep their levels conserved in plant tissue.

We further demonstrate that CK metabolism in *L. maculans* is active and can process exogenously supplied free CK bases very rapidly within the 1st hour after treatment (**Figure 2** and Supplementary Figure 1). While iP was metabolized predominantly into iPRMP, zeatins were converted mostly into their *O*-glucosides. Furthermore, *c*Z was metabolized more efficiently than *t*Z or iP as the levels of *c*Z*O*G were at least 10-fold higher than those of *t*Z*O*G or iPRMP, the main metabolites after feeding with *t*Z and iP, respectively. We showed, that both the metabolisations of iP into iPRMP, or *c*Z into *c*Z*O*G, are active processes and do not occur as spontaneous conversions without the presence of *L. maculans* or in killed mycelium (Supplementary Figure 2). In higher plants, CK inactivation may be achieved by the activity of *N*-glucosyltransferases (Hou et al., 2004), and, in case of zeatins, also by the activity of *O*-glucosyltransferases (Jin et al., 2013). Experimental data demonstrating the presence of these enzymes in fungi are missing. Our results suggest that *L. maculans* disposes of *O*-glucosyltransferase activity able to metabolize *c*Z and *t*Z (**Figure 2** and Supplementary Figure 2). Plant *O*-glucosyltransferases recognize CK substrate in a highly specific manner and specific enzymes for the glucosylation of *t*Z (Z*O*G; Martin et al., 1999) and *c*Z exist (*c*Z*O*G; Martin et al., 2001). Therefore, distinct *O*-glucosyltransferases may be present in *L. maculans*. *O*-glycosylated CKs in plants can be further easily converted to active free bases by β-glucosidases. Given these facts, *O*-glucosides are assumed to be inactive, or only weakly active, storage CK forms important for CK homeostasis (Mok and Mok, 2001). In *L. maculans*, the formation of both *t*Z and *c*Z *O*-glucosides following free base feeding was transitory (**Figure 2**). Besides, the mycelium contained only low levels of glucosides compared to free bases (**Figure 1C**). It seems therefore improbable that *O*-glucosides would function in *L. maculans* as stable, inactive CK-storage forms, analogous to that in plants. In addition, *N*-glucosylation activity is of weak importance in *L. maculans*. Only minute amounts of *N*-glucosides were present in the mycelium (**Figure 1A**). Accordingly, *N*-glucosides were not formed by *L. maculans* from CK bases within 24 h after feeding with any of the tested CKs (**Figure 2** and Supplementary Figure 1), although it cannot be excluded that *N*-glucosylation occurs with longer kinetics.

All exogenously feeded CK bases were partially converted to *t*Z and its levels remained stable within the 24-h time span studied (**Figure 2**). Based on our data, we do not know whether *L. maculans* possesses mechanisms to decrease the CK levels, especially *t*Z, that seem to accumulate in mycelium. The formation of *t*Z from iP indicates the presence of a hydroxylating enzyme. Hydroxylation represents a key step in *t*Z biosynthesis and is catalyzed by cytochrome P450 monooxygenases CYP735A1 and CYP735A2 in Arabidopsis (Takei et al., 2004). The presence of CK-specific P450 monooxygenase has been reported in *C. purpurea*, where deletion of cpp450 led to the loss of hydroxylated CKs and an increase in iP (Hinsch et al., 2015). This enzyme remains to be identified in *L. maculans*. On the other hand, our data strongly suggest the occurrence of zeatin *cis*–*trans* isomerase activity in *L. maculans* mycelium catalyzing *t*Z and *t*Z*O*G formation after *c*Z supply (**Figure 3**). The existence of zeatin *cis*–*trans* isomerase within the plant kingdom is rather controversial. Previously, zeatin *cis*–*trans* isomerase activity was reported in enzyme extract from immature *Phaseolus vulgaris* seeds (Bassil et al., 1993). Since then, studies supporting the existence of isomerase are very scarce, a gene encoding the hypothetical isomerase has not yet been found, and feeding experiments with radiolabeled precursors showed a distinct origin of the isoprenoid side chain in *t*Z and *c*Z in Arabidopsis (Kasahara et al., 2004). Further, only very low [potato; (Suttle and Banowetz, 2000)] or no [maize; (Yonekura-Sakakibara et al., 2004); tobacco, oat; (Gajdošová et al., 2011)] *in vivo* interconversions of zeatin stereoisomers have been reported in plants. For fungi, Behr et al. (2012) demonstrated that feeding of *C. graminicola* culture with *c*Z led to a substantial increase in *t*Z levels, which corresponds well with our findings in *L. maculans*. Similarly to our data, *C*. *graminicola* did not catalyze the reverse *t*Z to *c*Z conversion. Besides the isomerase catalyzed reaction, a non-enzymatic zeatin *cis*–*trans* conversion possibly takes place in *L. maculans* i*n vitro* culture in our conditions (**Figure 3**), in accordance with findings in *P. vulgaris* (Bassil et al., 1993). However, it cannot account for the whole zeatin *cis*–*trans* conversion, as the amount of *t*Z formed was only minute in boiled fungal culture. We also exclude the possibility that a *t*Z contamination in the feeded commercial *c*Z would be the sole source of *t*Z in our setup, given the amounts of detected *t*Z and *t*Z*O*G after *c*Z supply (**Figure 2**). The other direction of conversion via isomerase was not exhaustively investigated, as *trans*–*cis* conversion occurred with a lower intensity than the reverse *cis*–*trans* reaction.

### Cytokinin Oxidase/Dehydrogenases in Fungi?

Cytokinin oxidase/dehydrogenases catalyze the irreversible cleavage of the *N*^6^-side chain of isoprenoid CKs, the activity of which provides the key downregulation of their content in some organisms (Frébort et al., 2011). Besides higher plants, CKX activity has also been reported in a few lower organisms such as the mosses *Funaria hygrometrica* (Gerhäuser and Bopp, 1990) and *Physcomitrella patens* (von Schwartzenberg et al., 2007), the slime mold *Dictyostelium discoideum* (Armstrong and Firtel, 1989), and the yeast *Saccharomyces cerevisiae* (Van Kast and Laten, 1987), although the presence of endogenous CKX in the two latter organisms is doubtful (Schmülling et al., 2003). Additionally, *CKX* genes have been identified in several prokaryotic organisms, such as the bacteria *Rhodococcus fascians* (Pertry et al., 2010), and the presence of homologous genes has also been found in cyanobacterial genomes [e.g., of *Nostoc* and *Anabaena*; reviewed in (Schmülling et al., 2003; Frébort et al., 2011; Spíchal, 2012)]. No report on CKX activity in fungi has been available up to now. In our study, a significant activity of CKX to degrade iP was found in *L. maculans* mycelium using an *in vitro* radioisotope assay (**Figure 5**). In our hands, CKX activity does not apparently participate in the degradation of *cis*- and *trans*-zeatins, or does so only to a limited extent. This corresponds to the reported CKX substrate specificity in numerous higher plant tissues in which both *c*Z and *t*Z appear to be much poorer CKX substrates than iP (Armstrong, 1994). The capacity of CKX to degrade iP, *t*Z, and *c*Z also correlates well with the profile of accumulation of these CK forms in *L. maculans* mycelium in feeding assays (**Figure 4**). While applied iP was progressively degraded, zeatin isomers accumulated and their levels remained stable during the 24-h period. Interestingly, the CKX activity in *L. maculans* mycelium was pH dependent, with considerably higher values detected in the more alkaline buffer. This corresponds to the high pH optimum of CKX enzymes reported in multiple species of higher plants (Kamínek and Armstrong, 1990; Motyka et al., 2003; Gaudinová et al., 2005). Our data indicate that degradation of iP and its derivatives may represent an important pathway of CK metabolism in *L. maculans.* According to our knowledge, this is the first report demonstrating CKX activity in the fungal kingdom. Nevertheless, while the detected CKX activity in *L. maculans* mycelium for [^3^H] iP reached 64 pmol Ade mg^-1^ protein h^-1^ in the TAPS-NaOH buffer (pH 8.5), the plant protein extracts in analogous *in vitro* assays exhibited CKX activity one or two orders of magnitude higher. The CKX activity of *L. maculans* is thus considerably weaker than in plants and it is a question whether it can account for the entire clearance of iP. Given the fact, that zeatins are not degraded by the CKX activity in *L. maculans* and the fungus has probably tools to regulate these prevalent CK derivatives, the existence of other, as yet undescribed mechanisms of CK degradation in fungi may be envisaged.

### Role of Fungal IPTs

In plants, separate biosynthetic pathways ensure biosynthesis and an initial prenylation step of iP- and *t*Z-type CK derivatives on the one side (via adenylate IPT), and *c*Z-type derivatives on the other [via tRNA-IPT, (Sakakibara, 2006)]. The independence of both pathways results from their distinct origin and the localization of the isoprenoid side chains of iP- and *t*Z-type CKs (methylerythritol phosphate pathway in plastids) and *c*Z types (mevalonate pathway in cytosol) (Kasahara et al., 2004). Except from Archaea, tRNA-IPTs have been found in all organisms (Miyawaki et al., 2006). Based on the orthology, the *L. maculans* genome harbors only one copy of IPT, LmIPT, which is homologous to tRNA-IPTs (**Figure 6**). In various organisms, a deletion of tRNA-IPT leads to an abolished prenylation of tRNA, and a subsequent decrease or lack of *c*Z, such as: in Arabidopsis plants deficient in tRNA-IPTs, AtIPT2 and AtIPT9 (Miyawaki et al., 2006); in moss lacking PpIPT1 (Lindner et al., 2014); or in yeast lacking *Mod5* IPT (Dihanich et al., 1987). In our study, silenced LmIPT mutants exhibited a significant reduction in free *c*Z to about half their respective amount in the wild type (**Figures 7A,B**), indicating some function for LmIPT in *c*Z synthesis. Interestingly, the silencing of LmIPT caused increased levels of iP-type CKs and thus the total CK content was not decreased (**Figures 7C,D**). Recently it has been reported that CptRNA-IPT of *C. purpurea* is responsible for the entire *c*Z production (Hinsch et al., 2016). Accordingly, the knocking out of tRNA-IPT of *M. oryzae* (named CKS1 for Cytokinin Synthesis 1) totally abolished *c*Z production (Chanclud et al., 2016). The decrease of *c*Z levels due to the silencing of LmIPT is mild compared to these knockout studies but it may be caused, at least partly, by the low efficiency of the silencing method, currently used as the main tool for functional genetics in *L. maculans*. The successful knockout of tRNA-IPT in *M. oryzae* led to the complete loss of CK production, indicating that the formation of iP-type CKs is tRNA-IPT dependent in this fungus (Chanclud et al., 2016). Similarly, a double knockout of two sole IPT enzymes, tRNA-IPT and IPT-LOG, in *C. purpurea* led to a CK deficiency (Hinsch et al., 2016). No other gene with a clear IPT homology exists in *L. maculans*. Whether the whole CK production in this fungus depends on its sole *LmIPT* gene, or whether an IPT-independent pathway takes place, needs to be further elucidated using knockout mutants.

Taking advantage of knockout CK-deficient IPT mutants in fungi, it has been shown that CKs play an important role for the full virulence of *C. purpurea* on rye (Hinsch et al., 2016), and even of the non-tumor-forming pathogen *M. oryzae* on rice (Chanclud et al., 2016). Similarly, CKs are associated with virulence of *U. maydis* infection on maize (Morrison et al., 2015). In *L. maculans*, the decrease in *LmIPT* observed in our study did not considerably affect its virulence (Supplementary Figure 4C). It was proposed that the CKs of *M. oryzae* function as effectors to inhibit host defenses and deregulate nutrient distribution in rice (Chanclud et al., 2016). Whether the CKs of *L. maculans* may exert a similar role in its dicotyledonous host *B. napus* remains to be investigated.

### Role of Adenosine Kinase in CK Metabolism

Adenosine kinase is an enzyme involved in general purine metabolism that can also convert the adenine ring of CK ribosides to the corresponding nucleotides. AKs have been identified in animals, plants, and the prokaryote *Mycobacterium tuberculosis* (Long et al., 2003). Encoded AK proteins are of a length 324 – 348 aa, highly conserved among species and even kingdoms, sharing ∼85% identity. In Arabidopsis and tobacco, two and four functional AK isoforms (ADKs) are present, respectively (Moffatt et al., 2000; Kwade et al., 2005). Biochemical analyses have often shown that plant extracts or ADKs exhibit a higher affinity for adenosine than for CK ribosides (Chen and Eckert, 1977; Moffatt et al., 2000). However, AtADK1/2 silenced plants exhibited elevated CK riboside levels, showing that AKs of Arabidopsis play an important role in CK homeostasis *in vivo* (Schoor et al., 2011). An isoform ADK2S from *Nicotiana tabacum* exhibits a higher affinity to iPR and *t*ZR than to adenosine (Kwade et al., 2005). Little is also known about the AKs in fungi or other microorganisms. The genomes of *Dothideomycetes* fungi, including *L. maculans*, contain only one AK isoform (**Figure 8**). Fungal orthologs share ∼48% of their identity with plants. We show in this study that LmAK is involved in the conversion of CK ribosides into riboside 5′monophosphates (**Figures 9C,D** and Supplementary Table 1). The s.LmAK fungus was also impaired in its capacity to process exogenous iP into iPRMP, indicating that this metabolic step is at least partly dependent on LmAK (**Figure 9D**). In addition, s.LmAK lines were dramatically affected in *c*Z levels, which increased fivefold compared to the wild-type, showing the involvement of LmAK in CK metabolism (**Figure 9B**).

Other studies on microorganisms show the induction of *MoAK* due to cold stress in *M. oryzae* (Li et al., 2014), or the involvement of AK in extracellular polysaccharide production, cell motility, and virulence in *Xanthomonas campestris* (Lu et al., 2009). It was proposed that AK from *Saccharomyces cerevisiae* would be primarily implicated in the recycling of adenosine produced by the methylation cycle (Lecoq et al., 2001), but the involvement of microbial AKs in CK metabolism has not been studied. Our study is therefore the first reported function of fungal AK in CK metabolism. In plants, CKs can be converted to nucleotides by another pathway requiring adenine phosphoribosyltransferase (APT). Arabidopsis APT1 catalyzes the CK conversion from free bases to nucleotides (Zhang et al., 2013). The importance of AK and APT enzymes in this CK recycling varies according to species. For example, assays with radiolabelled CKs in *Physcomitrella* show that it is AK, but not APT, which is mainly responsible for the conversion of iP-riboside CK forms to the nucleotides (von Schwartzenberg et al., 1998). As no knowledge on the role of APT in fungi is available so far, it cannot be excluded that LmAPT also plays a role in CK recycling in *L. maculans*.

Adenosine kinase is considered to exert an essential function in eukaryotes. Arabidopsis plants deficient or silenced in AK have exhibited stunted growth, associated with decreased fertility or embryo lethality (Moffatt et al., 2002; Schoor et al., 2011). Our data show that LmAK is markedly required for a proper fungal growth (**Figures 10C,D** and Supplementary Figure 6), hyphae development (**Figure 10C** and Supplementary Figure 6B) and virulence on *B. napus* plants (**Figures 10A,B**). In additional to these CK-related cell processes, AK deficiency affects adenosine metabolism. In plants, adenosine is mainly produced by the hydrolysis of *S*-adenosyl-L-homocysteine (SAH) and recycled to adenine nucleotides by an adenosine salvage pathway requiring AK activity. SAH is involved in the methylation cycle of *S*-adenosyl-L-methionine (SAM), which controls methylations of pectin, lignin or DNA (Moffatt et al., 2000). An increased level of adenosine due to AK deficiency leads to SAH accumulation, further inhibiting the SAM recycling and transmethylation reactions (Moffatt et al., 2002). The *c*Z increase in s.LmAK mutants thus may be linked to a disrupted adenosine salvage pathway. Therefore, AK obviously exerts a broad-spectrum role, even in fungi, and is required for proper fungal growth, development and virulence.

### Different Roles of iP and Zeatins?

Different behaviors of iP and zeatins were observed throughout this study in *L. maculans*. Endogenous *t*Z and *c*Z levels remained stable over time, both in the mycelium and in the medium, after feeding with exogenous *t*Z and *c*Z, respectively, whereas endogenous iP was quickly eliminated after adding exogenous iP (**Figures 2, 4**). *L. maculans* also displayed the CKX activity capable of the degradation of iP but not zeatins (**Figure 5**). Furthermore, iP-type derivatives were not formed after feeding with zeatins, while feeding with all three CK bases led to an increase in both *t*Z and *c*Z (**Figure 2**). Furthermore, the silencing of LmIPT and LmAK affected mainly the *c*Z levels in *L. maculans* (**Figures 7B,D, 9B**). Based on these data, we hypothesize that iP might function as an active CK in *L. maculans* mycelium, or at least its levels are carefully controlled. Although iP is a minor CK in *Physcomitrella* compared to *c*Z, it represents the biologically active form inducing cell differentiation and growth limitation (Lindner et al., 2014). Additionally, iP and *t*Z displayed the strongest bud-inducing activity in *Physcomitrella* (von Schwartzenberg et al., 2007). Also, in many dicotyledonous plants, iP- and *t*Z-type CKs are the most active (Spíchal, 2012). The role of CKs in fungal physiological processes was previously mainly studied in mycorrhizal fungi. For instance, it was reported that CKs promote *in vitro* branching in ectomycorrhiza fungi (Barker and Tagu, 2000) and influence membrane transport (LeJohn and Stevenson, 1973). Similarly, CKs affect sexual reproduction (Elliott, 1967). Nevertheless the role of CKs in filamentous fungi still remains unclear. Silenced LmIPT mutants did not display altered growth nor morphology *in vitro* (Supplementary Figures 4A,B). Besides, no effect of the supplied CKs on the naturally occurring CKs of *L. maculans* and on the *in vitro* growth of *L. maculans* was observed (Supplementary Figure 3A). Only a higher concentration of BA and kinetin inhibited conidial growth (Supplementary Figure 3B). Different studies on the mutants of filamentous fungi with null CK production did not show distinctive growth impairment (Chanclud et al., 2016; Hinsch et al., 2016), though *M. oryzae* mutants were less tolerant to oxidative stress *in vitro* (Chanclud et al., 2016). Interestingly, the knockout of the hydroxylating enzyme cpp450, which led to an increase in iP and decrease in *t*Z, caused a hyper-sporulating phenotype in *C. purpurea* (Hinsch et al., 2015). In many fungi, *c*Z is a prevalent CK, including *L. maculans* (**Figures 1A,B**). In plants, it is believed to be mainly released with tRNA breakdown, which is a process well linked to photosynthesis during seed germination, or to protein breakdown during plant senescence (Schäfer et al., 2015). According to our data, the levels of CKs, and mainly the *c*Z types, increased with the age of *L. maculans* (**Figures 1A,B**). Accordingly, high levels of *c*Z in s.LmAK mutants (**Figure 9B**) might be due to the early onset of senescing events caused by the alteration of adenosine metabolism. It may be envisaged that the ageing and senescence of fungi may be correlated with increased *c*Z levels in a similar way to that in plants.

Taken together, this work shows that *L. maculans* can produce CKs *in vitro* and *in planta* and extends our knowledge on CK metabolism in fungi. New components of CK metabolism in fungi were described, such as AK being involved in the recycling of CKs into the pool of nucleotides, *cis*–*trans* isomerase contributing to *t*Z formation, and CKX playing an important role in CK degradation. Based on the data obtained in this study, a model illustrating and linking all these CK metabolic pathways in *L. maculans* has been proposed (**Figure 11**). Characterization of the new components in CK metabolism of *L. maculans* suggests some similarities with, but also differences from, plant CK metabolism. One can expect that the growing knowledge on CK metabolism in fungi draws the attention to many exciting questions, such as, for example: What are the pathways degrading CKs in fungi? What are the biological roles of the distinct CK forms in fungi? Do they work as environmental/developmental signals? How are these molecules perceived and which signaling pathways are used?

## Author Contributions

Conceived and designed the experiments: LT, VM, PD, VS, and LB. Performed the experiments: LT, MB, VM, PD, and LZ. Analyzed the data: LT, MB, VM, and PD. Wrote manuscript: LT, VM, PD, LB, and MN. All authors have read and approved the final manuscript.

## Conflict of Interest Statement

The authors declare that the research was conducted in the absence of any commercial or financial relationships that could be construed as a potential conflict of interest.

## Abbreviations

AK: adenosine kinase
CK: cytokinin
CKX: cytokinin oxidase/dehydrogenase
*c*Z: *cis*-zeatin
*c*Z7G: *cis*-zeatin 7-glucoside
*c*Z*O*G: *cis*-zeatin *O*-glucoside
*c*ZR: *cis*-zeatin 9-riboside
*c*ZRMP: *cis*-zeatin 9-riboside-5′-monophosphate
*c*ZR*O*G: *cis*-zeatin 9-riboside *O*-glucoside
DHZ: dihydrozeatin
DHZ7G: dihydrozeatin 7-glucoside
iP: *N^6^*-(Δ^2^-isopentenyl)adenine
iP7G: *N^6^*-(Δ^2^-isopentenyl)adenine 7-glucoside
iP9G: *N^6^*-(Δ^2^-isopentenyl)adenine 9-glucoside
iPR: *N^6^*-(Δ^2^-isopentenyl)adenine 9-riboside
iPRMP: *N^6^*-(Δ^2^-isopentenyl)adenine 9-riboside-5′-monophosphate
IPT: isopentenyltransferase
*t*Z: *trans*-zeatin
*t*Z7G: – *trans*-zeatin 7-glucoside
*t*Z9G: *trans*-zeatin 9-glucoside
*t*Z*O*G: – *trans*-zeatin *O*-glucoside
*t*ZR: *trans*-zeatin 9-riboside
*t*ZRMP: *trans*-zeatin 9-riboside-5′-monophosphate
