# Supplementary material for: Cytokinin Metabolism of Pathogenic Fungus Leptosphaeria maculans Involves Isopentenyltransferase, Adenosine Kinase and Cytokinin Oxidase/Dehydrogenase
Source: Front Microbiol. 2017 Jul 21;8:1374. doi: 10.3389/fmicb.2017.01374 (PMC5521058; doi:10.3389/fmicb.2017.01374)
Supplement: Supplementary file 1 [file Table_1.PDF]

Supplementary Table 1. Cytokinin metabolism in the silenced s.LmAK-105 mutant.

Content of cytokinin metabolites, adenine and adenosine in wild-type and silenced s.LmAK-105 transformant in non-treated cultures (-) or 1 h after treatment with iP 1 µM (+ iP). Data represent mean values ± SE (pmol g^{-1} dry weight, DW). The percentage of alterations compared to the wild-type are calculated with significant differences marked (*, *P*<0.05).

**CK metabolite**	**-**			**+ iP (1 hpt)**		
	**JN3**	**s.*Lm*AK-105**	**[%]**	**JN3**	**s.*Lm*AK-105**	**[%]**
free base						
iP	11.51 ± 4.11	14.31 ± 4.83	124	10813.65 ± 390.50	12058.55 ± 598.47	112
*t*Z	0.89 ± 3.74	3.74 ± 2.35	421	96.81 ± 4.40	90.33 ± 2.48	93
*c*Z	4.58 ± 0.49	26.66 ± 1.92	582*	15.89 ± 3.09	45.52 ± 4.76	286*
DHZ	nd	nd		nd	nd	
riboside						
iPR	0.70 ± 0.37	1.98 ± 0.19	282	1.32 ± 0.59	2.73 ± 0.29	207
*t*ZR	0.18 ± 0.15	1.82 ± 0.35	999*	nd	0.70 ± 0.30	-
*c*ZR	1.01 ± 0.48	8.01 ± 0.81	796*	0.81 ± 0.36	7.43 ± 0.18	916*
DHZR	0.30 ± 0.13	0.23 ± 0.03		0.08 ± 0.07	nd	
riboside monophosphate						
iPRMP	0.32 ± 0.17	0.14 ± 0.11	43	16.94 ± 1.91	7.15 ± 0.95	42*
*t*ZRMP	0.33 ± 0.27	0.24 ± 0.20	72	0.21 ± 0.17	nd	-
*c*ZRMP	0.75 ± 0.31	0.24 ± 0.20	32	0.53 ± 0.22	0.17 ± 0.14	32
DHZRMP	0.74 ± 0.30	nd	-	0.23 ± 0.19	nd	-
adenine	1073 ± 83	3551 ± 259	331*	912 ± 201	3473 ± 54	381*
adenosine	762 ± 169	851 ± 123	112	1190 ± 101	585 ± 54	49*
