# Supplementary figures and images for: Cytokinin Metabolism of Pathogenic Fungus Leptosphaeria maculans Involves Isopentenyltransferase, Adenosine Kinase and Cytokinin Oxidase/Dehydrogenase
Source: Front Microbiol. 2017 Jul 21;8:1374. doi: 10.3389/fmicb.2017.01374 (PMC5521058; doi:10.3389/fmicb.2017.01374)

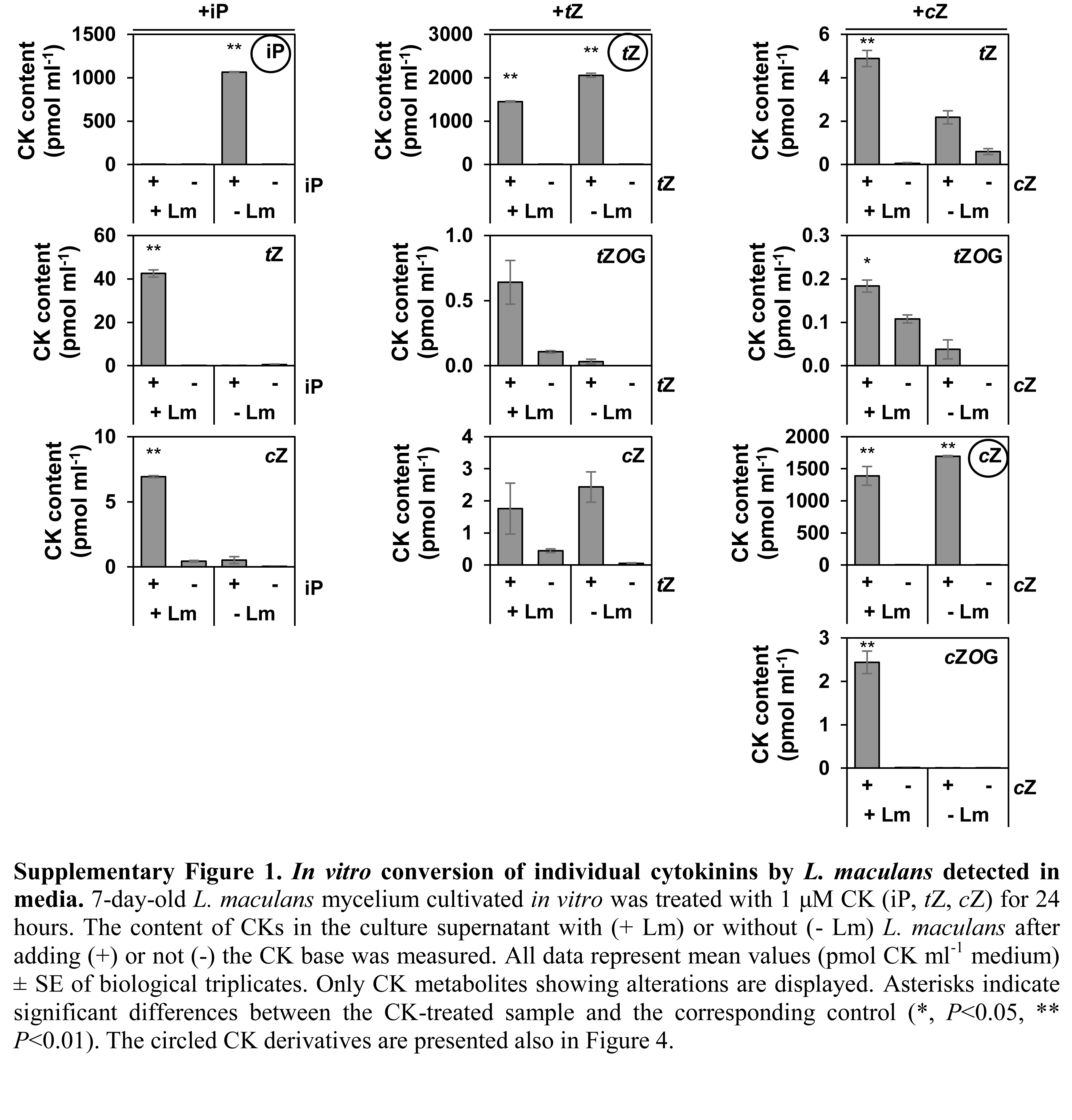

Supplement: Supplementary file 2 [file Image_1.TIF]

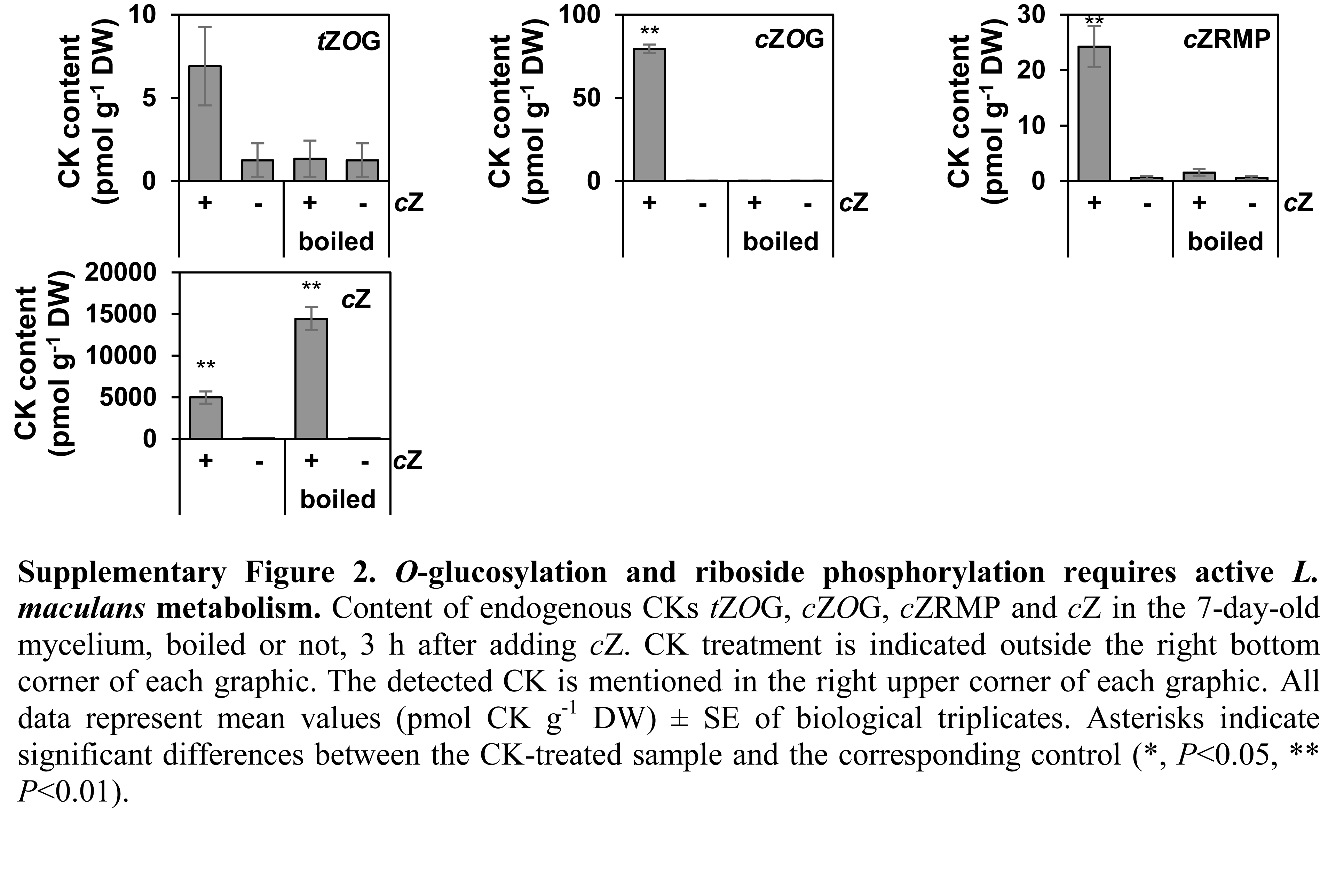

Supplement: Supplementary file 3 [file Image_2.tif]

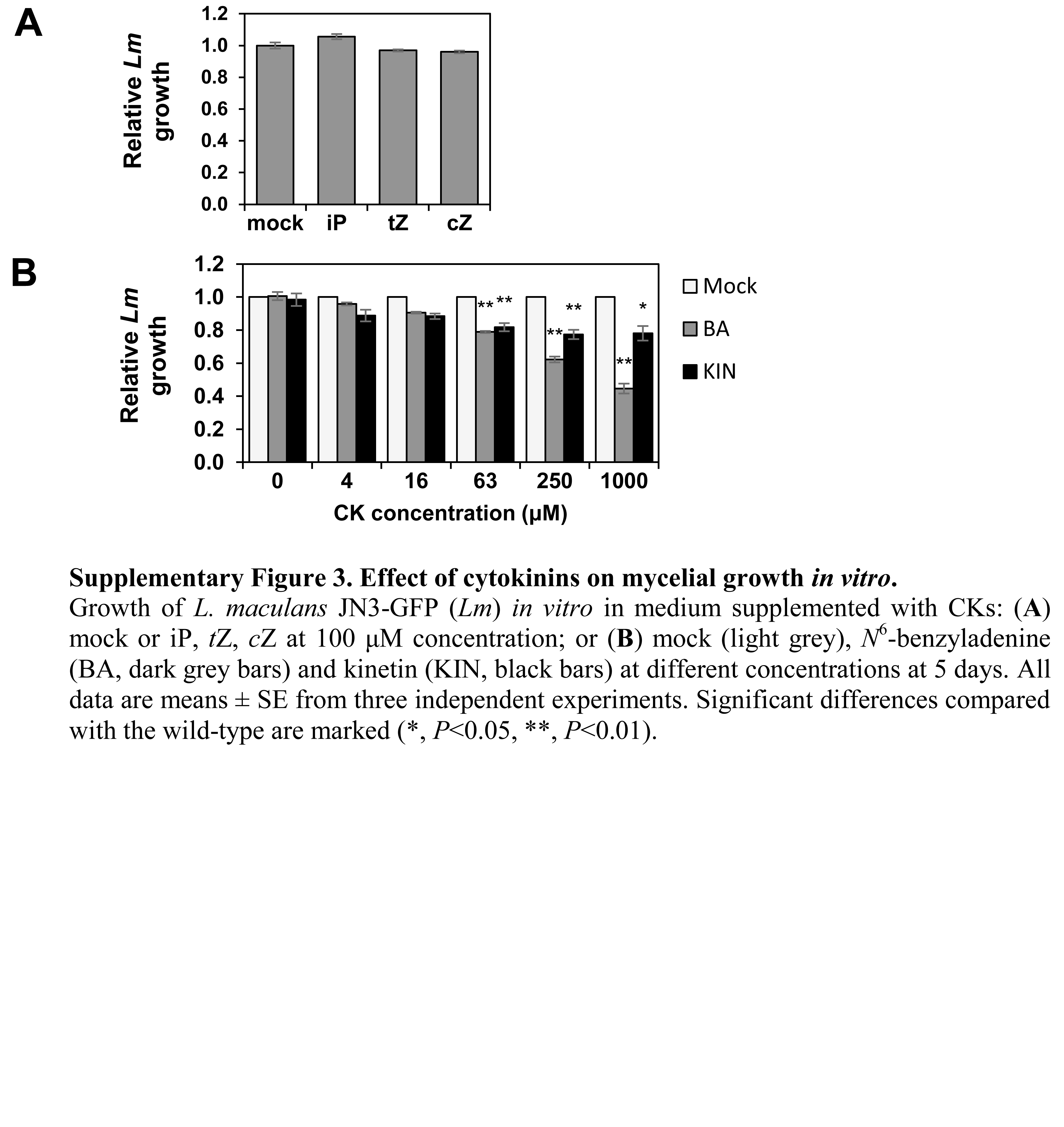

Supplement: Supplementary file 4 [file Image_3.TIF]

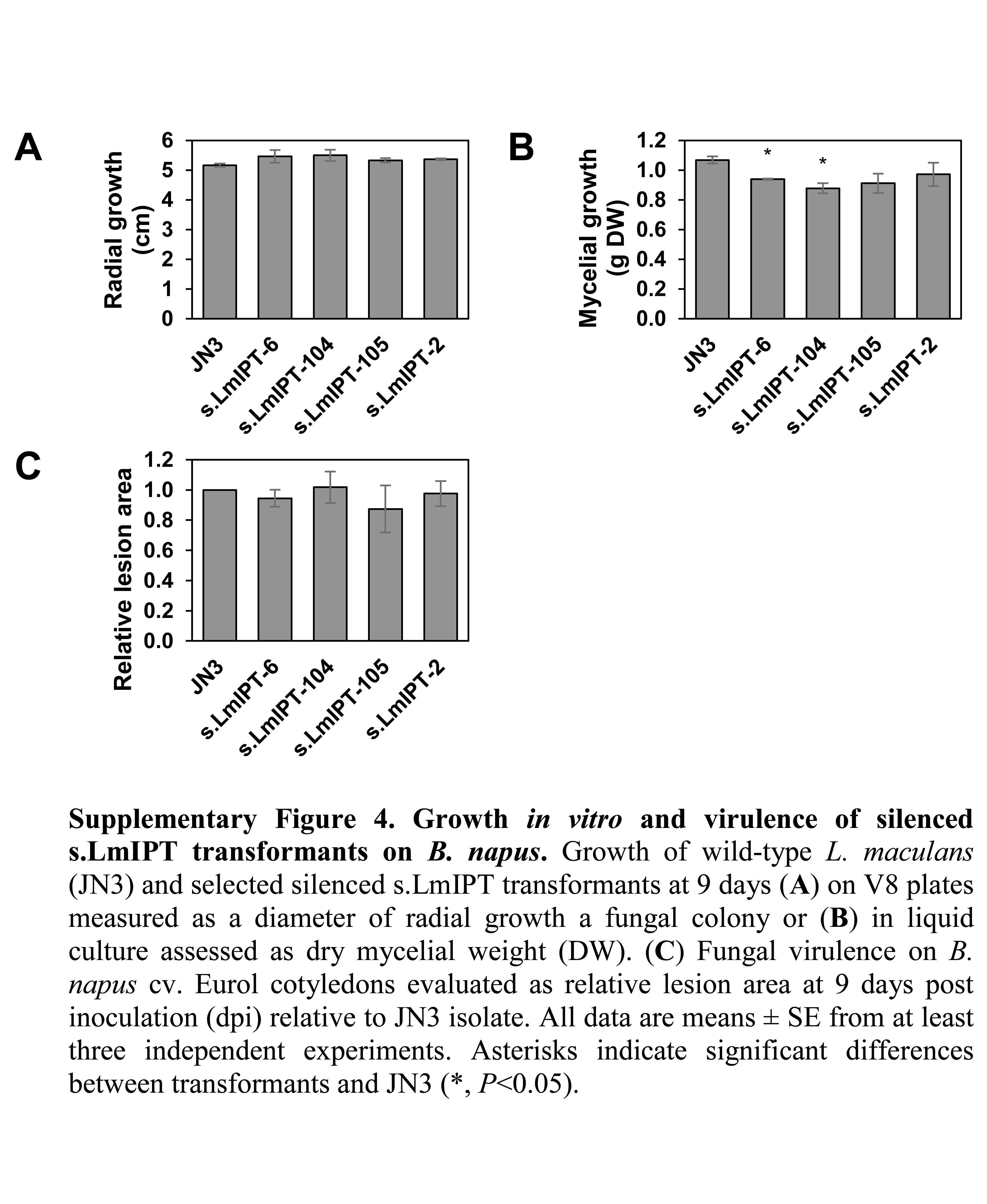

Supplement: Supplementary file 5 [file Image_4.TIF]

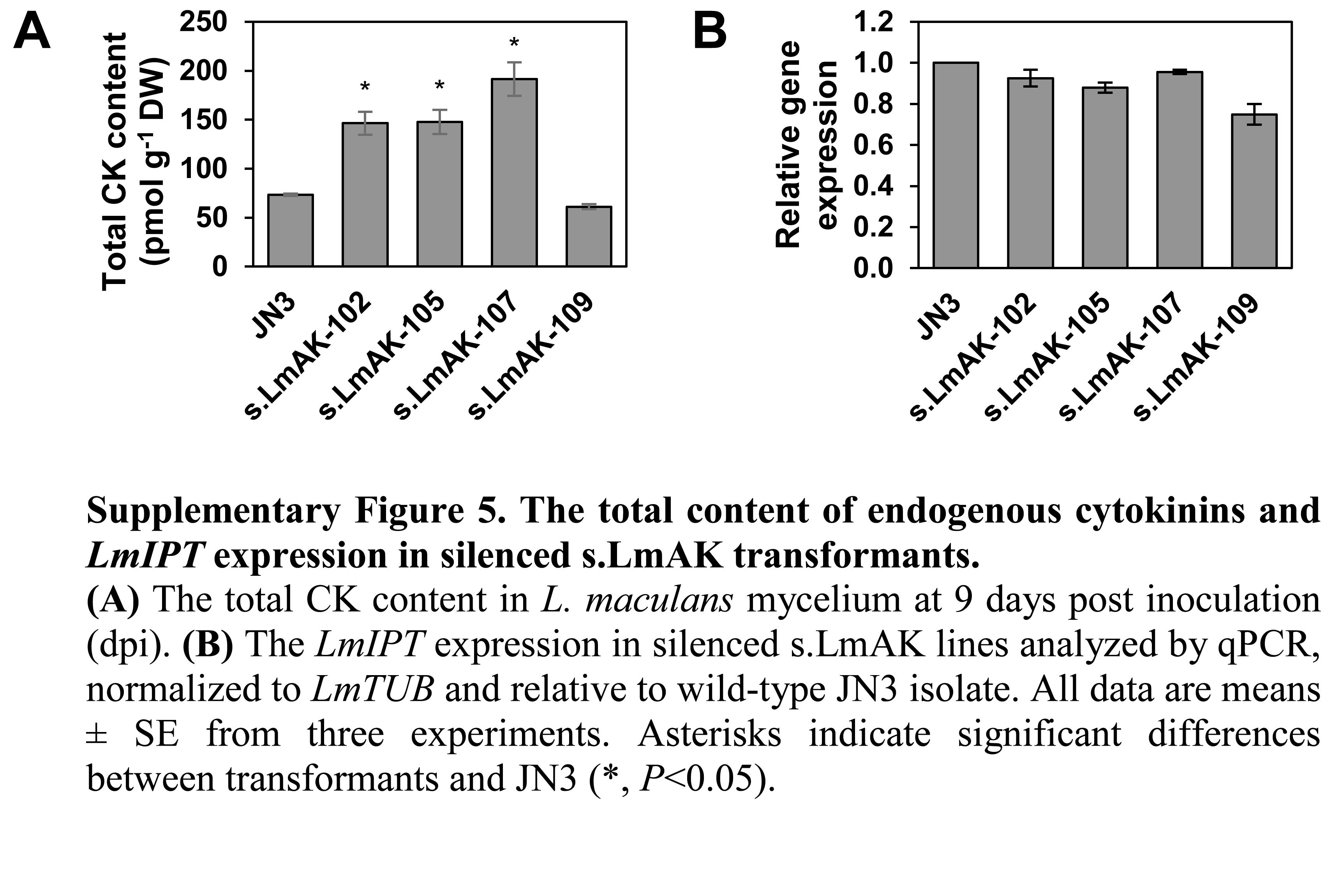

Supplement: Supplementary file 6 [file Image_5.TIF]

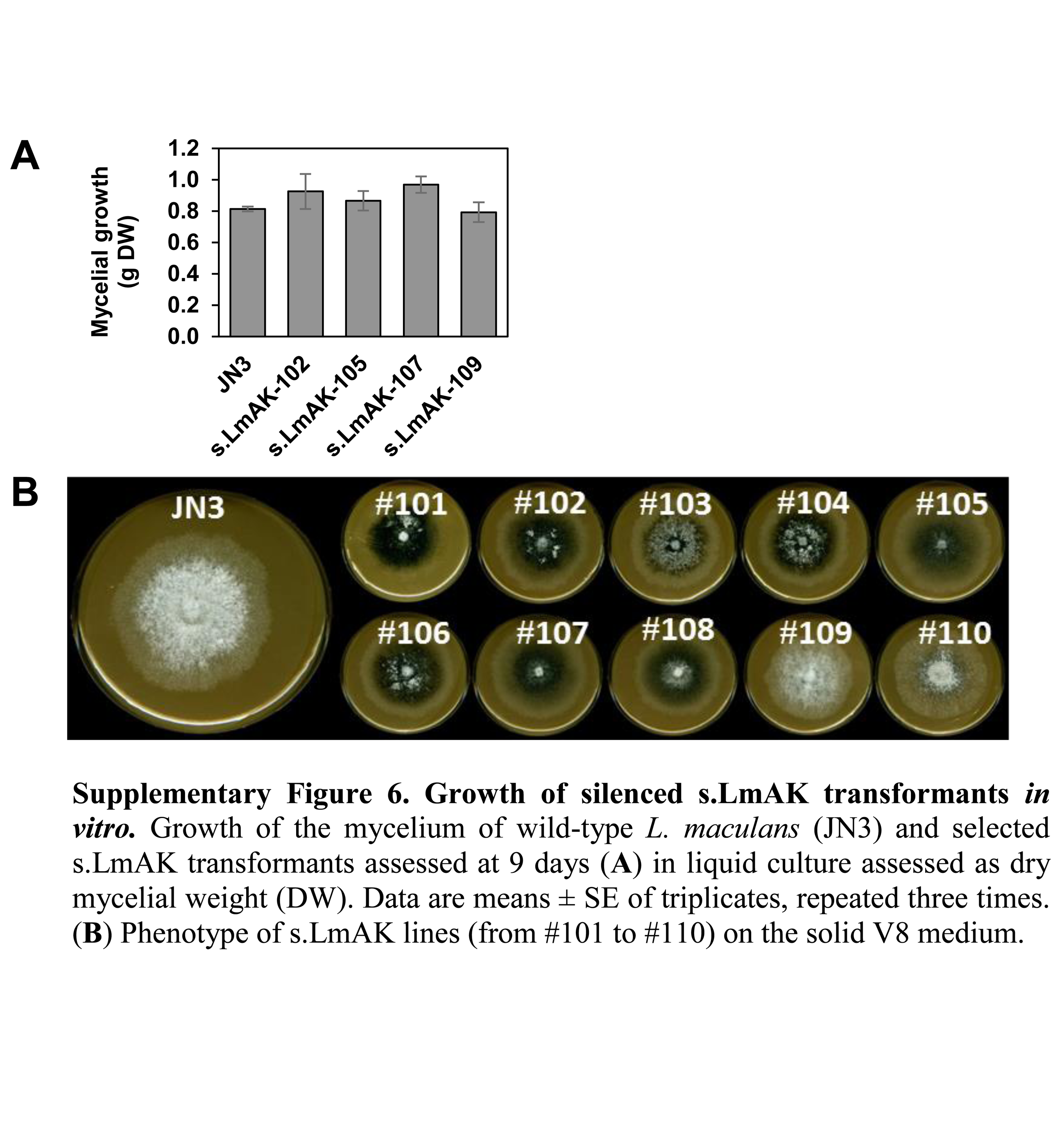

Supplement: Supplementary file 7 [file Image_6.TIF]
